# Antimicrobial Peptide Synergies for Fighting Infectious Diseases

**DOI:** 10.1002/advs.202300472

**Published:** 2023-07-05

**Authors:** Jessica T. Mhlongo, Ayman Y. Waddad, Fernando Albericio, Beatriz G. de la Torre

**Affiliations:** ^1^ KwaZulu‐Natal Research Innovation and Sequencing Platform (KRISP) School of Laboratory Medicine and Medical Sciences College of Health Sciences University of KwaZulu‐Natal Durban 4041 South Africa; ^2^ Peptide Science Laboratory School of Chemistry and Physics University of KwaZulu‐Natal Westville Durban 4000 South Africa; ^3^ CIBER‐BBN Networking Centre on Bioengineering Biomaterials and Nanomedicine and Department of Organic Chemistry University of Barcelona Barcelona 08028 Spain

**Keywords:** antibiotics, antimicrobial peptides (AMPs), Fractional Inhibitory Concentration Index (FICI), microbial resistance, synergy

## Abstract

Antimicrobial peptides (AMPs) are essential elements of thehost defense system. Characterized by heterogenous structures and broad‐spectrumaction, they are promising candidates for combating multidrug resistance. Thecombined use of AMPs with other antimicrobial agents provides a new arsenal ofdrugs with synergistic action, thereby overcoming the drawback of monotherapiesduring infections. AMPs kill microbes via pore formation, thus inhibitingintracellular functions. This mechanism of action by AMPs is an advantage overantibiotics as it hinders the development of drug resistance. The synergisticeffect of AMPs will allow the repurposing of conventional antimicrobials andenhance their clinical outcomes, reduce toxicity, and, most significantly,prevent the development of resistance. In this review, various synergies ofAMPs with antimicrobials and miscellaneous agents are discussed. The effect ofstructural diversity and chemical modification on AMP properties is firstaddressed and then different combinations that can lead to synergistic action,whether this combination is between AMPs and antimicrobials, or AMPs andmiscellaneous compounds, are attended. This review can serve as guidance whenredesigning and repurposing the use of AMPs in combination with other antimicrobialagents for enhanced clinical outcomes.

## Graphical abstract

Combination of antimicrobial peptides (AMPs) with other antimicrobial agents provides a new arsenal of drugs with synergistic action. The mechanism of action by AMPs is an advantage over antibiotics as it hinders the development of drug resistance. The synergistic effect of AMPs allows the repurposing of conventional antimicrobials, enhancing their clinical outcomes, reducing toxicity, and preventing the development of resistance.

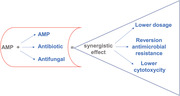

## 1 Introduction

Antibiotics are the most successful form of chemotherapy and essential medical interventions developed in the 20th century and possibly over the entire history of medicine. Antibiotics have saved countless human lives daily since the discovery of penicillin. However, their efficacy is threatened by the evolution of resistance in the organisms they are meant to kill. According to the 2019 report issued by the Centre for Disease Control and Prevention (CDC) in the USA, antibiotic resistance is one of the most predominant threats to public health, with more than 2.8 million humans infected and 35 000 deaths per year caused by direct infection by antibiotic‐resistance microorganisms.^[^ ^1^ ^]^ The World Health Organization (WHO) envisions 10 million deaths from antimicrobial resistance every 12 months until 2050.^[^ ^2^ ^]^ In this context, and to address the threat posed by antimicrobial resistance, in 2021, the CDC and more than 19 institutions in 38 nations launched the global Antimicrobial Resistance Laboratory and Response Network .^[^ ^3^ ^]^

The mechanisms of antimicrobial resistance have been reported.^[^ ^4^ ^]^ Most traditional antibiotics inhibit or kill microorganisms via a site‐specific binding mechanism and, as a result, they disrupt the metabolism and proliferation of the microorganisms.^[^ ^5^ ^]^ Hence, rapid development of antimicrobial resistance observed either through mutations on the binding site or alternate on the structure of the antibiotics, thus causing a loss in drug activity.^[^ ^6^ ^]^ Moreover, elevated efflux pump activity and reduction in membrane permeability lead to a lower intracellular antibiotic concentration, which is considered one of the key mechanisms of drug‐resistance in microorganisms.^[^ ^7^ ^]^ Subsequently, developing new strategies in addition to repurposing the use of conventional antimicrobials are urgently needed to fight the development of resistance.^[^ ^8^ ^]^ Given that some of the principal pharmaceutical companies closed down their antibiotic research departments because of unmet financial objectives, attention should now focus on the discovery of new classes of antimicrobial agents.^[^ ^9^ ^]^ In addition, the continued Coronavirus diseases (COVID‐19) pandemic has increased the incidence of antimicrobial resistance and reduced efforts devoted to the development of new antimicrobial agents.^[^ ^10^ ^]^

The antimicrobial peptides (AMPs), also known as host defense peptides, are present in most of the alive organisms for protecting the host again pathogens.^[^ ^11^ ^]^ Furthermore, they also display immunomodulatory properties. In this regard, AMPs have been identified as promising candidates to develop new drugs against resistant microorganisms.^[^ ^12^ ^]^ This has been translated into a large number of studies and in the entrance of several AMPs in clinical trials. Specially it is important the explosion on the number of clinical trials.^[^ ^13^ ^]^

Many studies shown that the majority of the AMPs inhibit or eliminate microbes through a membrane‐active mechanism rather than site‐specific binding or interference with bacterial metabolism.^[^ ^14^ ^]^ This mode of action points to their capacity to overcome the resistance related to traditional antimicrobial agents.^[^ ^15^ ^]^ However, given the capacity of microbes to rapidly develop resistance, new strategies are needed to limit resistance to AMPs.^[^ ^15^, ^16^ ^]^ Here we review the concurrent use of AMPs with different AMPs and antimicrobial agents to achieve synergistic action, wherein the combined antimicrobial effect is greater than the sum of either treatment alone.

## 2 Structural Diversity of AMPs

**Figure 1:**
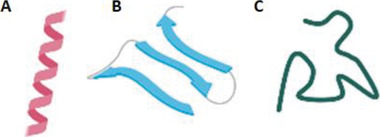
Secondary structures of AMPs: A) *α*‐helix, B) *β*‐sheet, and C) extended.

AMPs are ubiquitous and highly diverse, thereby making their classification very difficult. Although they can be categorized on the basis of their source, interest spectrum, ribosomal or nonribosomal origin, and biosynthetic mechanism, they are generally grouped in function of their structural properties. In this regard, there are three main structural classes, namely, i) *α*‐helical, ii) *β*‐sheet, and iii) extended AMP (**Figure** **1**).^[^ ^17^ ^]^ However, also are families of APM that combine structures of *α*‐helix and *β*‐sheet as the case of defensins (excluding *α*‐defensin) and presenting cyclic and other sophisticated structures (bacteriocins,^[^ ^18^ ^]^ *θ*‐defensins,^[^ ^19^ ^]^ cyclotides^[^ ^20^ ^]^).

### 2.1 *α*‐Helical AMPs

AMPs of this structural family are rich in positive‐charged residues and usually do not contain Cys. Whilst in aqueous solvents they are unstructured, their folding into amphiphilic *α*‐helix occurs in the presence of model or natural membranes. Once the peptide is structured, the hydrophilic part that contains a large number of polar amino acids faces the aqueous surface, and the counterpart incorporates the majority of hydrophobic residues.^[^ ^17b^ ^]^ By contrast, cationic peptides can bind electrostatically to the negatively charged membrane and, at a certain ratio of peptide to lipid, they can intrude the membrane and form transmembrane pores, thereby destabilizing the membrane—a process that leads to depolarization and cell death.

Representative examples of this group are the families of Cecropins,^[^ ^21^ ^]^ Magainins,^[^ ^22^ ^]^ Cathelicidins,^[^ ^23^ ^]^ and Spiderines.^[^ ^24^ ^]^

### 2.2 *β*‐Sheet AMPs

*β*‐Sheet peptides are another major class of AMPs. They are characterized by the presence of more than one *β*‐strand stabilized by hydrogen bonds, disulfide linkages, or even homodetic cyclic structures. Although, *β*‐sheets also act disrupting the bacterial membrane, in contrast to the *α*‐helical AMPs, they do not suffer an important conformational change when interacting with membranes because of their well stabilized structures by the disulfide bridges.

In this AMPs group are included the *α*‐defensins,^[^ ^25^ ^]^ and other peptides such as lactoferricin B,^[^ ^26^ ^]^ protegrin‐1,^[^ ^27^ ^]^ polymephusin and tachyplesin,^[^ ^28^ ^]^ and gomesin.^[^ ^29^ ^]^

### 2.3 Extended AMPs

Less common AMPs are extended peptides with a predominance of one or two amino acids (e.g., Gly, Arg, Pro, Trp, or His). Extended AMPs have no well‐defined structure as they lack typical secondary structure. However, due to the presence of a high proportion of those amino acids, these peptides adopt novel folds.^[^ ^30^ ^]^ These peptides commonly adopt extended structures when they interact with membranes. These structures are stabilized by hydrogen bonding and van der Waals forces with lipids rather than the intrapeptide interactions observed in other groups.^[^ ^30^, ^31^ ^]^

Prominent examples of extended AMPs include indolicidin, which is dominated by Pro, Trp, and Arg,^[^ ^32^ ^]^ and human histatins, which are His‐rich compounds.^[^ ^33^ ^]^ Another particularly interesting subclass are Pro‐ and Arg‐rich AMPs, which include several examples from insects, such as drosocin,^[^ ^34^ ^]^ pyrrhocoricin,^[^ ^35^ ^]^ and honeybee apidaecins, which contain 34 amino acids, of which 30% are Pro,^[^ ^36^ ^]^ while from mammalian is PR‐39, which is rich in Pro–Arg residues, although it belongs to the cathelicidin family.^[^ ^37^ ^]^

## 3 Chemical Modifications of AMPs and Their Impact on AMP Properties

For drug development, the synthesis and chemical modification of short AMPs is an efficient tool to reduce the elevated cost of preparing large peptides. Bioinspired by natural AMPs, this modification seeks to reduce peptide toxicity, enhance solubility, and improve antimicrobial activity.^[^ ^38^ ^]^ In particular, bactenecin is a cationic peptide, composed of 12 amino acids, which inhibit the growth of some Gram‐negative bacteria. Several bactenecin analogs result in increased activity against both Gram‐negative and Gram‐positive bacteria.^[^ ^39^ ^]^ Also, synthetic AMPs have high purity and are highly selective toward bacterial cells because they are engineered with specific sequence modifications,^[^ ^40^ ^]^ such as cyclization, scanning, amino acid substitution, electric charge, hydrophobicity, and even hybridization.

The stability of AMPs to proteases is a key for their activity.^[^ ^41^ ^]^ The antimicrobial activity and proteolytic stability of these peptides can also be improved by the substitution of l‐amino acids with d‐amino acids. In this regard, the clinical therapeutic outcomes can be enhanced by developing protease‐resistant AMPs.^[^ ^42^ ^]^

**Figure 2:**
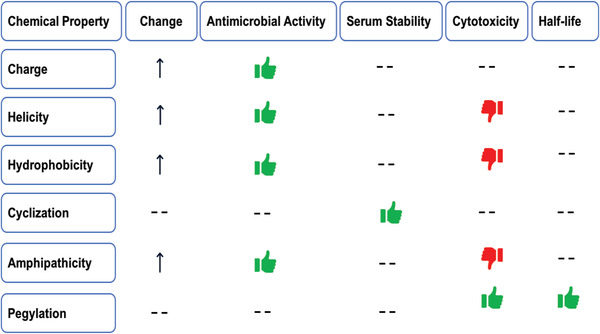
Effect of chemical modification of AMPs on some properties.

The properties of polyethylene glycol (PEG) make it an ideal candidate as a carrier for peptides and a tool to overcome the challenge of the short half‐life associated with AMPs.^[^ ^43^ ^]^ PEG can be attached to the N or C terminus of the peptides or to the peptide sequence through nucleophilic amino acids.^[^ ^44^ ^]^ PEG–peptide conjugates evince the properties of high‐water solubility, high mobility in solution, reduced renal clearance, and significantly reduced immunogenicity of peptide therapeutics (**Figure** **2**)).^[^ ^45^ ^]^

## 4 Mechanism of Action of AMPs

Many AMPs have evolved to be able to kill bacteria with minimal toxicity to host cells. This selectively is due to the structural differences between prokaryotic microorganisms and eukaryotic cells. Both Gram‐positive and Gram‐negative bacteria carry a negative charge in their surface membranes as a result of the presence of teichoic and lipoteichoic acid on the outer membrane of the former and lipopolysaccharide (LPS) and phosphatidyleglycerol on that of the latter. Therefore, AMPs will interact electrostatically with the negatively charged bacterial cell membrane. On contrary, the zero net charge of the mammalian cell membrane prevents electrostatic interaction with positively charged AMPs.^[^ ^46^ ^]^

### 4.1 Pore‐Formation Mechanism

Upon reaching a given concentration, the AMP may self‐assemble on the membrane and form a pore lead to control ion flow across the membrane followed by cell lysis and death.^[^ ^23^, ^47^ ^]^ The mechanism by which AMPs form pores in bacterial membranes is still unclear. However, the following have been proposed: i) the barrel‐stave model, ii) the carpet mechanism, and (iii) toroidal pore formation.

**Figure 3:**
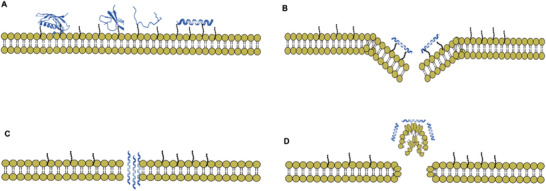
Selected mechanisms of membrane disruption with A) accumulation of peptide on the surface, B) toroidal pore formation, C) barrel‐stave pore formation, and D) the carpet model.

All these models result in cell death through the formation of holes in the membrane, thereby leading to the impairment of transmembrane potential.^[^ ^47d^ ^]^ In this regard, the effective combined and synergistic action of AMPs in killing multidrug‐resistant bacteria is attributed to their membrane permeabilization capacity (**Figure** **3**).^[^ ^48^ ^]^

### 4.2 Other Mechanisms of Action of AMPs

AMPs do not cause membrane permeabilization and bacterial death only. There is increasing evidence that these peptides translocate into the cytoplasm where they target and inhibit a variety of key cellular processes, including cell wall synthesis, enzyme synthesis, nucleic acid synthesis,^[^ ^49^ ^]^ and protein production, such as human cathelicidin LL‐37 and bactenecin, without causing extensive membrane damage.^[^ ^47^, ^50^ ^]^

The AMP buforin II and other *α*‐helical peptides, such as derivatives of pleurocidin, translocate dermaseptin, drosocin, pyrrhocoricin, apidaecin, *β*‐sheet human defensin, and HNP‐1^[^ ^51^ ^]^ isolated from frog skin across the bacterial membrane without causing permeabilization. They also bind to both DNA and RNA within the cytoplasm of *Escherichia coli*. On the other hand, bovine peptide indolicidin^[^ ^49a^ ^]^ has caused inhibition of DNA and RNA synthesis at their Minimum Inhibitory Concentration (MICs) without destabilizing the membrane of bacterial cells.

AMPs directly target the structural components of the cell wall, where they inhibit cell wall synthesis, thereby leading to the killing of both sensitive and resistant bacteria. The bacterially produced lantibiotics mersacidin and nisin inhibit the synthesis of peptidoglycan by interfering with the transglycosylation of lipid II methicillin‐resistant *Staphylococcus aureus* (MRSA) strain. Although the antibiotic vancomycin targeted the same biosynthetic process, cases of resistance were reported. On contrary, the action of mersacidin and nisin on distinct molecular moieties within lipid II confers activity against vancomycin‐resistant bacteria.^[^ ^52^ ^]^

In addition, protein synthesis is an important target of AMPs. The uptake of *E. coli* for tritiated leucine can be diminished by pleurocidin and dermaseptin, and the cells subjected to doses of indolicidin and PR‐39 show reduced protein synthesis.^[^ ^49^, ^53^ ^]^

Pro‐rich insect AMPs like pyrrhocidin play a key role in regulating cellular enzymatic activity. The heat‐shock protein DnaK binds to pyrrhocidin, thereby interrupting chaperone‐assisted protein folding. The Pro‐rich insect AMPs pyrrhocidin, apidaecin, and drosocin specifically inhibit Dnak ATPase activity, thus enhancing the concentration of misfolded proteins and inducing cell death.^[^ ^54^ ^]^

The use of more than one mechanism of action by AMPs is also observed during the infection state. The ability of these peptides to interact and bind to the negatively charged molecules within the cytoplasm, such as nucleic acids, points to their involvement in the destabilization of the cell membrane, as well as in the inhibition of intracellular targets.

## 5 Microbial Resistance to AMPs

AMPs are less likely to promote the development of drug resistance than current antibiotics. In this regard, AMPs act mainly on cell membranes, thus hindering pathogen development of resistance since these microorganisms are then required to modify membrane components, a process that is difficult and carries a metabolic cost for cells. However, bacteria are commonly exposed to AMPs in their natural habitats and, as a result, have developed resistance. Given that AMPs exert their action on the cell membrane and are also involved in the identification of specific proteins, the possibility for genetic mutation and bacterial resistance arises.^[^ ^17^, ^55^ ^]^

Some reports document the rare presence of bacterial resistance to AMPs.^[^ ^56^ ^]^ However, at the correct concentration and suitable combination with antibiotics, AMPs emerge as promising antimicrobials to combat multiresistant bacteria.^[^ ^17^, ^57^ ^]^ Moreover, the reduction of antimicrobial‐resistant bacteria by exploiting the synergistic action of certain antimicrobial agents can provide desirable antimicrobial activity at lower drug concentrations.

## 6 Defeating Antimicrobial Resistance by AMP Synergies

Drug combination therapies are applied in many diseases, in the case of infectious diseases is supposed that could be a strategy to eliminate resistant strains, or even delay the evolution of drug resistance. Of special interest are the drug combinations that present a synergistic interaction, i.e., combinations of drugs that have an effect that is larger than the additive independent contribution of the individual drugs. This kind of interaction will reduce the dosage of individual drugs needed, and hence, diminish the undesired side effects.^[^ ^58^ ^]^

**Figure 4:**
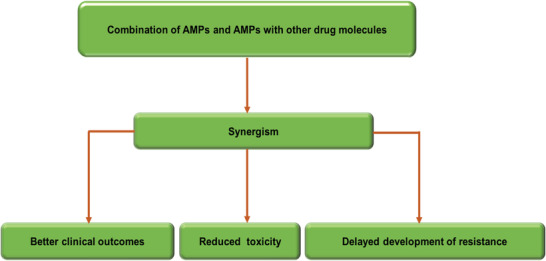
Expected benefits of AMPs when used in combination.

Although few papers were devoted to this topic in the 1990s, increasing attention is reflected by the growth in the number of publications dedicated to this topic in 2000. Indeed, since 2010, a considerable number have been published each year. In this regard, interest in drug synergies has grown in response to increasing antibiotic resistance. AMPs have demonstrated good synergy with other antimicrobial agents due to their pore formation in microbial membranes, thereby facilitating access of other antimicrobials and permitting the inhibition of DNA/RNA synthesis, protein synthesis, or cell wall synthesis.^[^ ^59^ ^]^ Strategies that harness the synergistic effect of antimicrobial compounds have immense capacity to increase the potency of treatment, reduce antibiotic resistance, and extend the lifetime of traditional antimicrobial agents.^[^ ^60^ ^]^ They might also be useful against the formation of biofilms on surfaces,^[^ ^61^ ^]^ consequently reducing potential toxicity and economic costs.^[^ ^59^ ^]^ This phenomena of synergism is of biological and clinical importance (**Figure** **4**).^[^ ^62^ ^]^

To quantify drug interactions is used the Fractional Inhibitory Concentration (FIC) Index. It is calculated by the addition of the individual FIC of each drug that is determined by dividing each drug's MIC when used in combination by each drug's MIC when used alone

(1) $$\mathrm{FIC}=\frac{{\mathrm{MIC}}_{A}^{\mathrm{Mixture}}}{{\mathrm{MIC}}_{A}^{\mathrm{Alone}}}+\frac{{\mathrm{MIC}}_{B}^{\mathrm{Mixture}}}{{\mathrm{MIC}}_{B}^{\mathrm{Alone}}}$$

The value obtained is used to categorize the interaction between two drugs tested in combination. The FIC values were defined as synergy for FCI < 0.5, additivity for 0.5 < FCI ≤ 1, indifference for 1 < FIC ≤ 2, and antagonism for FIC > 2.

From the following sections all the sequences corresponding to AMP mentioned in the text are listed in the Supporting Information by order of appearance.

### 6.1 AMP–AMP Synergism

#### 6.1.1 Magainins

**Figure 5:**
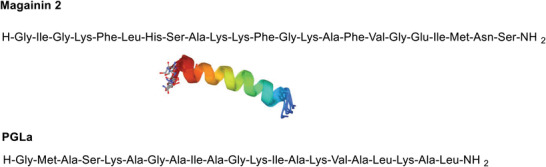
Amino acid sequences of magainin 2 and PGLa. Helical structure of magainins in DPC micelles obtained from Protein Data Bank (PDB).

Magainins are a class of AMPs that were discovered in the skin of the African clawed frog *Xenopus laevis*. It is assumed that they adopt an amphipathic *α*‐helical structure when they interact with membranes. The most widely studied members of this class^[^ ^22^ ^]^ are magainin 2^[^ ^63^ ^]^ and peptidyl‐glycyl‐leucine carboxyamide (PGLa) (**Figure** **5**).^[^ ^64^ ^]^ Both peptides show antimicrobial activity against Gram‐positive and Gram‐negative bacteria and against fungi, they induce osmotic lysis of protozoa, and they have high selective toxicity.^[^ ^22^, ^65^ ^]^

Interestingly, when used in combination, magainin 2 and PGLa show greater activity than when used alone. A 1:1 ratio of magainin 2 and PGLa shows marked functional synergism (approximately tenfold enhancement of membrane disruption and antibacterial activity). The reported synergistic action of the magainin 2–PGLa peptide combination was reported during membrane depolarizing studies.^[^ ^66^ ^]^ It was later found that this combination is synergistic in diverse systems.^[^ ^67^ ^]^ Using several antimicrobials and biophysical methods, the synergism of PGLa/magainin 2 on a range of lipid membranes was addressed by various authors, and recently reviewed by Bechinger et al.^[^ ^50^, ^68^ ^]^

The characteristics of the magainin 2 and PGLa peptide, such as charge, hydrophobicity, helicity, sequence, length, and amphiphilicity, appear to be crucial for their mechanism of action. Magainin 2 and PGLa carry several positive charges (nominal charge +4 to +5), which are essential for initial binding to the anionic bacterial membrane surface, a factor that allows discrimination between bacterial and host cell membranes.^[^ ^69^ ^]^ According to Williams et al., magainin 2 binds to the surface of liposomes made of negatively charged lipids but without spontaneously penetrating the bilayer. Upon binding to liposomes, magainin 2 forms a helix with an average length of less than 20 Å. PGLa and magainin 2 interact with liposomes in a synergistic way that enhances the helix content of one or both peptides and allows them to penetrate the bilayer more easily.^[^ ^70^ ^]^

Hydrophobicity is needed for insertion into and disruption of the target membrane.^[^ ^69^ ^]^ According to Strandberg et al., the antimicrobial activity of magainin 2 is consistently improved when hydrophobicity (replacing Gly with Ala or CF_3_‐Bpg (bicyclopent‐[1.1.1]‐1‐ylglycine)) is increased on the hydrophobic face of the amphiphilic helix.^[^ ^71^ ^]^ This observation reflects the importance of the hydrophobic residues for the antimicrobial properties of magainin 2. However, the synergy between magainin 2 and PGLa does not depend on the overall hydrophobicity of the former.^[^ ^71^ ^]^

The amphiphilic *α*‐helix structure of magainin 2 disrupts the membrane by forming channels (consisting of complexes of peptide monomers)^[^ ^22^, ^72^ ^]^ or pores, thereby causing membrane instability, leakage of K+, and osmotic lysis. Matsuzaki et al. suggested that the rate of pore formation is slower for magainin 2 than for PGLa. However, the openings of the former are more stable.^[^ ^73^ ^]^ Therefore, synergism between these two peptides arises from the formation of a potent heterosupramolecular complex, which is characterized by rapid pore formation and moderate pore stability, as well as the formation of supramolecular structures along the membrane interface.^[^ ^73^, ^74^ ^]^

A cross‐linking study in membranes showed that these two peptides form a parallel heterodimer that is likely aligned parallel to one another, as the CC‐ and NN‐terminally linked heterodimers are the most active compared with the monomeric species, thus implying that the observed synergism is due to heterodimer formation.^[^ ^75^ ^]^ According to Nishida et al., the activity of a chemically fixed heterodimer is like that of a 1:1 mixture of the two separate peptides, thus the synergy is due to the formation of a parallel heterodimer.^[^ ^76^ ^]^

The Hill coefficients determined from vesicle leakage data highlighted that the heterodimeric (magainin 2–PGLa) interactions were stronger than the homodimeric (PGLa–PGLa and magainin 2–magainin 2) ones. This observation was also reflected in the free energy of dimerization determined from oriented circular dichroism and quantitative solid‐state ^19^F NMR analysis.^[^ ^77^ ^]^ These heterodimers perturb membranes significantly more than the sum of the effects induced by each peptide alone.^[^ ^78^ ^]^ Simulation studies have revealed that aggregated peptides do not retain side‐by‐side heterodimeric structures but instead show anchoring between the C‐terminal groups of magainin 2 and PGLa, thereby allowing deeper insertion of PGLa into the bilayer.^[^ ^62^, ^79^ ^]^

The interaction involved in magainin 2–PGLa synergy does not include covalent bonding since the covalent heterodimers do not improve the capacity of PGLa/magainin 2 mixtures to interact synergistically. Therefore, more indirect interactions may play a part through modulations of the lipid packing, thereby leading to enhanced synergistic interaction.^[^ ^80^ ^]^ Recently, it has been shown that PGLa and magainin 2 form fibers at physiological conditions and that these fibers are somewhat less active in antimicrobial assays but maintain the synergy.^[^ ^81^ ^]^ A combination of experimental and computational techniques revealed the surface‐aligned fibril‐like structure of PGLa and magainin 2 that induces membrane fusion aggregates between membranes, which in turn cause membrane adhesion, fusion, and finally, the formation of a sponge phase.^[^ ^63^ ^]^

The orientation of PGLa and magainin 2 in membranes has been extensively studied using solid‐state NMR and has provided clues about the synergistic mechanism between the two peptides.^[^ ^82^ ^]^ The topological alignment of PGLa with respect to the membrane surface is affected by the detailed composition of the phospholipid membrane, its peptide‐to‐lipid ratio, the hydration level, and the presence of the other peptide.^[^ ^82^, ^83^ ^]^

PGLa and magainin 2 remain flat on the membrane surface in lipids with a negative spontaneous curvature. However, in lipids with a positive spontaneous curvature, only PGLa assumes a titled orientation but it inserts into the bilayer in a transmembrane alignment in the presence of magainin 2. By contrast, magainin 2 is only slightly titled or stays on the surface either alone or in the presence of PGLa. These observations indicate that curvature‐dependent helix orientation is a general feature of membrane‐bound peptides and that it might also influence their synergistic intermolecular interactions.^[^ ^82b^ ^]^ The lipid fatty acyl chain and the lipid head group composition have been reported to be of considerable importance for synergistic enhancement.^[^ ^81^ ^]^ In this context, for lipids with an intrinsic negative curvature, such as PE or PC/cholesterol, the pore‐forming activity of the individual peptides is reduced when compared to PC/PG membranes. This observation suggests that the membrane curvature has only a minor effect on PGLa realignment, while the important driving force of this realignment is the magainin 2‐induced disordering of fatty acyl chains.^[^ ^84^ ^]^

The synergic effect of the combination magainin 2–PGLa, in general is more pronounced for Gram‐negative bacterial cells, such as strains of *E. coli*,^[^ ^67^, ^73^ ^]^ even has been demonstrated that magainin 2–PGLa synergy also confers antifouling activity on surfaces and antibiofilm activity against *Pseudomonas aeruginosa*.^[^ ^61^ ^]^ However, it has also been studied in *S. aureus* and *Staphylococcus eperdemidis*.^[^ ^64^, ^76^, ^80^, ^84^ ^]^

**Figure 6:**
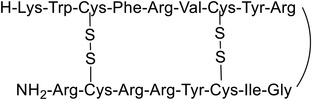
Amino acid sequence of Tachyplesin I. Cyclic *β*‐sheet peptide containing two disulfide bridges.

Magainin 2 has also been described to have a strong synergistic effect in combination with tachyplesin I against Gram‐negative and Gram‐positive bacteria. These two peptides, not only have different origin if not that are structurally different. Tachyplesin I is a cyclic *β*‐sheet peptide (**Figure** **6**) and its structure seems to be essential for the selective synergical effect. Although the mechanism is not clear it points out to be related to permeabilization of acidic phospholipid‐containing membranes.^[^ ^85^ ^]^

#### 6.1.2 Temporins

Temporins AMPs were first isolated from the European red frog *Rana temporaria* ^[^ ^86^ ^]^ however the family was extended to other AMPs produced by frogs around the world. They are short peptide in a range of 8 to 17 residues, being 13 the most common. Usually have a high content on Leu and Ile, only one or two positively charged amino acids, mainly Lys, and lack of Cys.^[^ ^87^ ^]^ As in the case of magainins, they adopt amphipathic *α*‐helical structures. These peptides are active mainly against Gram‐positive bacteria, fungi as *C. albicans*, and also showed leishmanicidal activity.^[^ ^86^, ^88^ ^]^ Additionally, some temporins have shown to act in human tumor cell lines, including human monoclonal leukemia (U‐937), human erythroleukemia (K‐562), and human cutaneous T Lymphoma (Hut‐78).^[^ ^89^ ^]^The mode of action of temporins suggests that they act directly on the membrane of the parasite by destroying its integrity, thereby making it difficult for the pathogen to develop resistance.^[^ ^90^ ^]^

**Figure 7:**
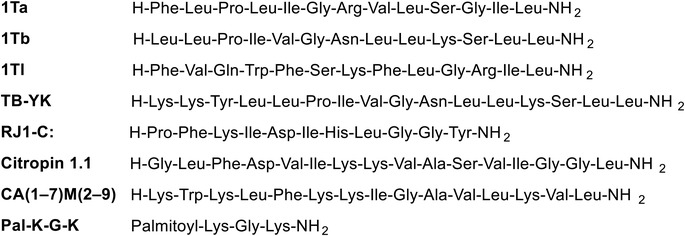
Amino acid sequence temporins and synergistic peptides.

The isoform temporins‐1Ta, 1 Tb, and 1Tl (**Figure** **7**) are among the most studied temporin peptides for their mechanism of action on both intact bacteria and artificial systems. Despite being selectively addressed to Gram‐positive bacteria, when isoforms 1Ta and 1 Tb are combined with temporin‐1Tl, each shows a synergistic effect against Gram‐negative bacteria.^[^ ^91^ ^]^ The rationale behind this behavior is that the major constituent of the outer membrane of Gram‐negative bacteria is LPS, which plays an important role in the activity of temporins. LPS induces the oligomerization of temporin‐1 Tb and prevents its translocation across the outer membrane, thereby reducing its activity on Gram‐negative bacteria. However, temporin‐1Tl has no oligomerization and shows greater activity.^[^ ^91^ ^]^ Thus, temporin 1Tl interacts synergistically with temporin 1Ta and 1 Tb by preventing their oligomerization in LPS, and promoting their translocation across the outer membrane into the cytoplasmic membrane.^[^ ^92^ ^]^ NMR and fluorescence spectroscopy have revealed the synergistic effect of the combined use of temporin‐1 Tb and ‐1Tl to overcome the LPS‐mediated barrier.^[^ ^93^ ^]^ The synergy of these two peptides may result from their structures, their proximity in the environment of LPS micelles, and changes in the structural states of LPS (**Table** **1**).^[^ ^93^ ^]^

**Table 1:** In vitro antimicrobial synergy between temporins and other AMPs

#	AMP 1	AMP 2	Organism	FICI	Refs.
1	Temporin‐1TI	Temporin‐1Tb	Lipopolysaccharide micelles	N/A	[93]
2	Temporin TB‐YK	Temporin A	*S. aureus* A170	0.5	[94]
3	Temporin TB‐YK	Temporin A	*S. aureus* A172	0.5	[94]
4	Temporin TB‐YK	Temporin A	*Salmonella enterica serovar Paratyphi*	0.4	[94]
5	CA(1–7)M(2–9)NH2	Temporin A	MRSA (ATCC 43300) MRSA 357426 MRSA 355872 MRSA 348839	0.25 0.38 0.5 0.26	[48]
6	CA(1–7)M(2–9)NH2	Citropin 1.1	MRSA (ATCC 43300) MRSA 357426 MRSA 355872 MRSA 348839	0.5 0.48 0.5 0.5	[48]
7	CA(1–7)M(2–9)NH2	Pal‐KGK‐NH2	MRSA (ATCC 43300) MRSA 348839	0.5 0.5	[48]
8	Pal‐KGK‐NH2	Temporin A	MRSA (ATCC 43300)	0.5	[48]
9	Pal‐KGK‐NH2	Citropin 1.1	MRSA 357426	0.38	[48]
10	Citropin 1.1	Temporin A	MRSA 357426 MRSA 355872	0.38 0.31	[48]

By a simple modification on temporin B sequence where the N‐terminal has been extended by the tripeptide KKY (Temporin TB‐YK) it is shown to act synergistically with temporin A (Figure 7). The mixture of both peptides had antimicrobial and anti‐inflammatory activity against Gram‐positive and Gram‐negative bacteria in vivo (**Figures** 2, 3, 4).^[^ ^94^ ^]^

**Table 2:** In vitro antimicrobial synergy between defensin and other AMPs

AMP^a)^	AMP^a)^	Organism	FICI	Refs.
HD 5	HD 6	*E. coli, S. aureus, E. aerogenes*, *and B. cereus*	N/A	[101, 107]
Cec6	Def4	*E. coli, M. luteus*	0.41	[108]
Cg‐Defh1	Cg‐Defh2	*M. lysodeikticus* CIP5345	0.50	[109]
Cg‐Defh2	Cg‐Defm	*M. lysodeikticus* CIP5345	0.37	[110]
Cg‐Defh2	Cg‐Defm	*E. coli* SBS 363	0.50	[110]
Cg‐Defh2	Cg‐Defm	*V. splendidus* LGP32 CIP 107715	0.50	[110]
Cg‐Defh1	Cg‐IgPrp	*M. lysodeikticus* CIP5345	0.35	[109]
Cg‐Defh1	Cg‐IgPrp	*E. coli* SBS 363	0.50	[109]
Cg‐Defh2	Cg‐IgPrp	*M. lysodeikticus* CIP5345	0.30	[109]
Cg‐Defh2	Cg‐IgPrp	*E. coli* SBS 363	0.28	[109]
Cg‐Defm	Cg‐IgPrp	*M. lysodeikticus* CIP5345	0.45	[110]
HNP‐1	HNP‐2	*S. mutans*	0.961	[103]
HNP‐1	HNP‐3	*S. mutans*	0.933	[103]
HNP‐2	HNP‐3	*S. mutans*	0.849	[103]
HNP‐1	HBD‐2	*S. mutans*	0.799	[103]
HNP‐1	HBD‐3	*S. mutans*	0.918	[103]
HNP‐2	HBD‐2	*S. mutans*	0.715	[103]
HNP‐3	HBD‐2	*S. mutans*	0.629	[103]
HNP‐3	HBD‐3	*S. mutans*	0.779	[103]
Cg‐Prp_22‐36_	Cg‐defensin	*M. lysodeikticus*	0.33	[111]
Cg‐Prp_26‐36_	Cg‐defensin	*M. lysodeikticus*	0.33	[111]
Cg‐Prp	Cg‐defensin	*E. coli*	0.29	[111]
Porcine beta‐defensin (PBD‐1)	PG‐3	*E. coli* and multidrug‐resistant *S. typhimurium* DT104	N/A	[112]
Porcine beta‐defensin (PBD‐1)	PR‐39	*E. coli* and multidrug‐resistant *S. typhimurium* DT104	N/A	[112]
Porcine beta‐defensin (PBD‐1)	PR‐26	*E. coli* and multidrug‐resistant *S. typhimurium* DT104	N/A	[112]
Human defensin	Rabbit defensin	*C. albicans*	N/A	[113]

^a)^ All sequences are listed in the Supporting Information.

**Table 3:** In vitro antimicrobial synergy between cathelicidin and other AMPs

AMP^a)^	AMP^a)^	Organism	FICI	Refs.
LL‐37	Lugdunin	*S. aureus*	N/A	[122]
LL‐37	Gallidermin	*S. aureus*	N/A	[122]
Human cathelicidin LL‐37	Human *β*‐defensin (1–3)	*P. gingivalis*	N/A	[123]
Cathelicidin‐related AMP	Onc72 Onc110 Onc112	*E. coli* ATCC 25922	0.48 0.30 0.30	[119]
Cathelicidin‐related AMP	Onc72 Onc110 Onc112	*E. coli* DSM 10233	0.32 0.20 0.29	[119]
Cathelicidin‐related AMP	Onc72 Onc110 Onc112	*E. coli* DSM 5922	0.43 0.27 0.30	[119]
Cathelicidin‐related AMP	Onc72 Onc110 Onc112	*K. pneumoniae* DSM 11678	0.28 0.22 0.29	[119]
Cathelicidin‐related AMP	Onc112	*S. enteritidis* ATCC 14028	0.49	[119]
Cathelicidin‐related AMP	Onc110	*P. aeruginosa* DSM 9644	0.41	[119]
Cathelicidin‐related AMP	Onc72 Onc110	*A. baumannii* DSM 30008	0.50 0.44	[119]
Cathelicidin‐related AMP	Api88 Api137	*E. coli* ATCC 25922	0.36 0.35	[119]
Cathelicidin‐related AMP	Api137	*E. coli* DSM 5923	0.50	[119]
Cathelicidin‐related AMP	Api88 Api134	*K. pneumoniae* DSM 11678	0.28	[119]
Cathelicidin‐related AMP	Api134	*K. pneumoniae* DSM 11678	0.39	[119]
Cathelicidin‐related AMP	Api137	*K. pneumoniae* DSM 11678	0.25	[119]
LL‐37	Human *β*‐ defensin‐3	Broad range of oral Gram‐positive and Gram‐negative bacteria	N/A	[124]
HNP‐1	LL‐37	*S. mutans*	0.586	[103]
HNP‐2	LL‐37	*S. mutans*	0.645	[103]
HNP‐3	LL‐37	*S. mutans*	0.587	[103]
Indolicin	LL‐37	*E. coli*		[118b]
Human cathelicidins (LL‐37)	Beta defensin‐2	Streptococcus	N/A	[125]
LL‐37	HBD‐2	*S. aureus*	N/A	[104]
Indolicin	LL‐37	*E. coli*	0.50	[121]
LL‐37	Protegrin 1	*P. aeruginosa*	0.31	[121]
LL‐37	Protegrin 1	*E. coli*	0.31	[121]
LL‐37	Protegrin 1	*E. faecalis*	0.32	[121]
LL‐37	Bactenecin	*E. faecalis*	0.50	[121]
Bactenecin	LL‐37	*P. aeruginosa*	0.50	[121]
Bactenecin	LL‐37	*E. coli*	0.50	[121]
Indolicin	Protegrin 1	*P. aeruginosa*	0.25	[121]
Indolicin	Protegrin 1	*E. coli*	0.25	[121]
Human defensin (HNP‐1)	LL‐37	*E. coli* and *S. aureus*	N/A	[105]
Guinea pig defensin	CAP11	*E. coli* and *S. aureus*	N/A	[105]

^a)^ All sequences are listed in the Supporting Information.

**Table 4:** In vitro antimicrobial synergy of lantibiotics and other AMPs

AMP^a)^	AMP^a)^	Organism	FICI	Refs.
Bsj*α*	Bsj*β*	Gram‐positive strains including methicillin‐resistant *S. aureus* and vancomycin‐resistant *Enterococci*	N/A	[129]
Lantibiotic Lch*α*	Lch*β*	*M. luteus* B1314, and *B. megaterium* VKM41	N/A	[131]
LtnA1	LtnA2	Lactococcal cells, liposomes	N/A	[130]

^a)^ All sequences are listed in the Supporting Information.

Temporin B and the royal jellein I chemically modified at the C terminal (RJ1‐C) (Figure 7) were used in combination and showed synergy against *Staphylococcus epidermidis*, displaying both antimicrobial and anti‐inflammatory activities.^[^ ^95^ ^]^ It is believed that being both from different origin and RJ1‐C forming *β*‐sheet are the reasons for this synergistic effect.

Recently, an study including temporin A and another temporin, citropin 1.1, and the synthetic peptides CA(1–7)M(2–9) and Pal‐KGK‐NH_2_ (Figure 7), has been carried out by doing different combinations 2 by 2 and testing against MRSA biofilms developed on polystyrene and medical devices. The results shown that the synergistic action of temporin A and CA(1–7)M(2–9)NH_2_ was stronger than that of temporin A and Pal‐KGK‐NH_2_ or other combination on the same MRSA strain.^[^ ^48^ ^]^ The combinations that show synergistic effects are shown in **Tables** 5, 6, 7, 8, 9, 10.

**Table 5:** In vitro antimicrobial synergy between bacteriocins and other AMPs

AMP^a)^	AMP^a)^	Organism	Refs.
Plantaricin J	Plantaricin K	Gram‐positive bacteria, including *M. luteus*, *L. plantarum*, and *S. epidermidis*	[140]
Plantaricin E	Plantaricin F	Gram‐positive *Staphylococci* strains	[139]
Cbnx	Cbny	C*. divergens* LV13 and *C. maltaromaticum* A9b	[146]
Enterocins 7A	7B	Not applicable	[18]
Thuricin D *α*	Thuricin D *β*	*C. difficile*	[147]
Enterocin L50A	Enterocin L50B	*L. brevis* and *P. damnosus*	[145]
Plantaricin alpha	Plantaricin beta	*L. sakei* strains NCDO 2714	[148]

^a)^ All sequences are listed in the Supporting Information.

**Table 6:** In vitro antimicrobial synergy between other AMPs

AMP^a)^	AMP^a)^	Organism	FICI	Refs.
Lugdunin	Dermicidin‐derived peptides	*S. aureus*	N/A	[122]
Bombinin	Bombinin h	*S. aureus*	0.375	[150]
Abaecin	Hymenoptaecin	*E. coli*	N/A	[155]
Protenectin	Protenectin (1–6)	Sodium dodecyl sulfate (SDS) micelles	N/A	[154b]
Feleucin‐BV1	Novel bombinin	*S. aureus* (NCTC 10788), *E. coli* (NCTC 10418), and *C. albicans* (NCPF 1467)	0.5	[151]
Gal 7	Gal 9	*S. enteriditis*	N/A	[156a]
Hepcidin	Moronecidin	*Y. enterocolitica*	0.5	[156c]
SMAP29	OaBac5mini	*E. coli*	0.31	[156b]
SMAP29	OaBac7.5mini	*E. coli*	0.31	[156b]
OaBac5mini	OaBac7.5mini	*E. coli*	0.31	[156b]

^a)^ All sequences are listed in the Supporting Information.

**Table 7:** In vitro antimicrobial synergy between AMPs and antibiotics

AMP^a)^	Antibiotic	Organism	FICI	Refs.
WLBU2	Minocycline Novobiocin	*Y. pestis* CO92 *Y. pestis* CO92 *F. tularensis* LVS	0.50 0.02 0.195	[164]
L12	Vancomycin Levofloxacin	*S. aureus* S26, S47, S49	0.188–0.375 0.125–0.500	[165]
D‐LL‐31	Ceftazidime	*B. pseudomallei* 1026b, H777, M10	0.125–0.188	[166]
LfcinB (20–25)_2_	Ciprofloxacin	*E. coli* 25922	0.09	[167]
LfcinB (20–25)_4_	Ciprofloxacin Vancomycin Vancomycin	*P. aeruginosa* *E. faecalis* 29212 *S. aureus* 29923	0.09 0.06 0.04	
LfcinB (21–25)_Pal_	Vancomycin	*E. faecalis* 29212 *S. aureus* 29923	0.14 0.01	
L11W	Penicillin Ampicillin Erythromycin	MDR *S. epidermidis*	0.312 0.281 0.281	[168]
L12W	Penicillin Ampicillin Erythromycin	MDR *S. epidermidis*	0.281 0.257 0.281	
I1WL5W	Penicillin Ampicillin Erythromycin Tetracycline	MDR *S. epidermidis*	0.281 0.258 0.281 0.282	
I4WL5W	Penicillin Ampicillin Erythromycin	MDR *S. epidermidis*	0.188 0.156 0.312	
Protegrin‐1	Colistin Fosfomycin Meropenem Tigecycline	MDR *K. pneumoniae*	0.182–0.500 0.094–0.500 0.251–0.500 0.252–0.500	[169]
SLAY‐P1	Vancomycin	Vancomycin‐resistant *Enterococci*	0.125	[170]
Melamine	Ciprofloxacin	Ciprofloxacin resistant *P. aeruginosa* 37	0.38	[171]
AamAP1‐Lysine	Levofloxacin Chloramphenicol Erythromycin Rifampicin	MRSA MDR *P. aeruginosa* MDR *P. aeruginosa* MRSA MRSA	0.103 0.36 0.36 0.204 0.128–0.203	[172]
UP5	Levofloxacin Rifampicin Ampicillin Chloramphenicol	*S. aureus* ATCC 29213 *S. aureus* ATCC 33591 *S. aureus* ATCC 43300 *S. aureus* ATCC 33591 *S. aureus* ATCC 43300 *P. aeruginosa* ATCC BAA‐2114 *S. aureus* ATCC 33591 *P. aeruginosa* ATCC BAA‐2114	0.11 0.13 0.25 0.13 0.45 0.46 0.27 0.46	
HYL (2,7,8,10,11,16,17,18,19,22,23)	Amoxycillin	*S. aureus*	0.17–0.50	[173]
HYL (1,4,19,25)	Tetracycline	*P. aeruginosa*	0.31–0.42	
HYL (1,2,4,7,8,9,10,11,12,14,15,16,17,18,19,20,21,22,23,24,25)	Rifampicin	*P. aeruginosa*	0.21–0.44	
CAMP11i	Kanamycin Rifampicin	*L. monocytogenes*	0.24 0.374	[174]
BA250‐C10	Tobramycin	*P. aeruginosa* KD491 *P. aeruginosa* LESB58 *P. aeruginosa* Clone C	0.375–0.5 0.5 0.5	[162]
I(LLKK)_2_I M(LLKK)_2_ M W(LLKK)_2_W	Rifampicin	*M. smegmatis*	0.5	[175]
Plectasin	Amoxacillin Penicillin Flucoxacillin Gentamicin Neomycin Amikacin	MSSA and MRSA	≤ 0.5	[176]
PL‐(5,18,26,29,31,32)	Imipenem Cefepime Levofloxacin Vancomycin	*P. aeruginosa* *K. pneumonia* *S. aureus* *S. epidermidis* *S. pneumonia* *E. coli* *S. aureus* *S. epidermidis* *S. pneumonia*	0.031–0.313 0.040–0.381 0.031–0.313 0.141–0.381	[177]
Plectasin NZ2114	Teicoplanin Dalbavancin	VanA‐type glycopeptide‐resistant *E. faecalis*	0.20 0.26	[178]
PMAP‐36	Gentamicin	*E. coli*	0.375	[179]
Coprisin	Ampicillin Vancomycin Chloramphenicol	*E. faecium* ATCC 19434 *S. aureus* ATCC 25923 *S. mutans* KCTC 3065 *E. coli O‐157* ATCC 43895 *E. coli* ATCC 25922 *P. aeruginosa* ATCC 27853 *E. faecium* ATCC 19434 *S. aureus* ATCC 25923 *S. mutans* KCTC 3065 *E. coli* O‐157 ATCC 43895 *E. coli* ATCC 25922 *P. aeruginosa* ATCC 27853 *E. coli* O‐157 ATCC 43895 *P. aeruginosa* ATCC 27853	0.375–0.500 0.250–0.500 0.500	[180]
CAMA	Azithromycin Daptomycin	MRSA ATCC 43300 MRSA ATCC 43300	0.500 0.375	[160]
Indolicin	Daptomycin	MRSA ATCC 43300	0.500	
Nisin	Daptomycin Linezolid Teichoplanin Ciprofloxacin	MRSA ATCC 43300	0.375 0.375 0.250–0.375 0.125–0.250	
Pleurocidin	Ampicillin Chloramphenicol Erythromycin	*S. aureus* ATCC 25923 *P. acnes* ATCC 6919 *E. coli* ATCC 25922 *E. coli* O‐157 ATCC 43895 *P. aeruginosa* ATCC 27853 *S. aureus* ATCC 25923 *E. faecium* ATCC 19434 *P. acnes* ATCC 6919 *E. coli* ATCC 25922 *E. coli* O‐157 ATCC 43895 *P. aeruginosa* ATCC 27853	0.375‐0.500 0.375–0.500 0.375–0.500	[181]
Protegrin 1	Aureomycin	EPEC O78K80	0.160	[182]
Cathelicidin‐BF	Aureomycin	EPEC O78K80	0.280	
D‐peptide‐A	Tetracycline Piperacillin Cefotaxime Chloramphenicol Rifampicin	MRSA, *P. aeruginosa* *P. aeruginosa* *P. aeruginosa* *P. aeruginosa* *P. aeruginosa*	0.500, 0.125 0.312 0.500 0.187 0.25	[183]
D‐peptide‐B	Tetracycline Piperacillin Cefotaxime Chloramphenicol Rifampicin	MRSA, *P. aeruginosa* *P. aeruginosa* *P. aeruginosa* MRSA, *P. aeruginosa* *P. aeruginosa*	0.500, 0.187 0.250 0.312 0.500, 0.187 0.125	
D‐peptide‐C	Tetracycline Piperacillin Ceftazidime Chloramphenicol	MRSA, *P. aeruginosa* *P. aeruginosa* MRSA *P. aeruginosa*	0.500 0.312 0.375 0.500	
D‐peptide‐D	Tetracycline Piperacillin Cefotaxime Chloramphenicol Rifampicin	MRSA, *P. aeruginosa* MRSA, *P. aeruginosa* *P. aeruginosa* MRSA, *P. aeruginosa* *P. aeruginosa*	0.312, 0.500 0.375 0.250 0.187 0.312	
Citropin 1.1	Clarithromycin Doxycycline Rifampicin	*R. equi*	0.312 0.385 0.385	[184]
Temporin A	Co‐amoxiclav Imipenem	*E. faecalis* ATCC 29212, *E. faecalis* ATCC 51299 *E. faecalis* ATCC 29212, *E. faecalis* ATCC 51299	0.312	
Deacylpolymyxin B	Rifampicin Erythromycin Fusidic acid Novobiocin	*E. coli* IH3080	0.030 0.130 0.040 0.040	[185]
KFFKFFKFF	Rifampicin Erythromycin Fusidic acid Novobiocin	*E. coli* IH3080	0.030 0.060 0.100 0.400	
IKFLKFLKFL	Rifampicin Erythromycin Fusidic acid Novobiocin	*E. coli* IH3080	0.060 0.130 0.100 0.130	
CKFKFKFKFC	Rifampicin Erythromycin Fusidic acid Novobiocin	*E. coli* IH3080	0.200 0.300 0.400 0.330	
KKKKKKFLFL	Rifampicin	*E. coli* IH3080	0.400	

^a)^ All sequences are listed in the Supporting Information.

**Table 8:** In vitro antimicrobial synergy between AMP and fluconazole

AMP^a)^	Antifungal	Organism	FICI	Refs.
LL‐37	Fluconazole	*C. auris* various strains	0.25–0.5	[206]
Cc‐GRP	Fluconazole	*F. solani*	N/A	[205]
DS6	Fluconazole	Clinical isolates of *C. tropicalis*	0.3	[204]
CaThi	Fluconazole	*Candida* species	N/A	[202]
*β*‐peptide	Fluconazole	*C. albicans*	0.5	[201]
KU4	Fluconazole	*C. albicans* SC5314	0.38	[197]
KU4	Fluconazole	*C. albicans* ATCC 90028	0.34	[197]
Upn‐lys6	Fluconazole	*C. albicans* SC5314	0.42	[197]
KABT‐AMP	Fluconazole	*C. albicans* SC5314	0.50	[197]
uperin 3.6	Fluconazole	*C. albicans* SC5314	0.50	[197]
IB‐367	Fluconazole	*T. mentagrophytes*, T*. rubrum* and *M. canis*	N/A	[196]
Hepcidin 20	Fluconazole	*C. glabrata*	0.5	[195]
PAL‐Lys‐Lys‐NH2	Fluconazole	*C. parapsilosis* 4796, 4882	0.48–0.50	[194]
PAL‐Lys‐Lys‐NH2	Fluconazole	*C. albicans ATCC90029, 4890*	0.48–0.50	[194]
PAL‐Lys‐Lys‐NH2	Fluconazole	*C. tropicalis* ATCC750, 4795	0.30–0.48	[194]
PAL‐Lys‐Lys‐NH2	Fluconazole	*C. krusei ATCC6258, 4684, 4153*	0.12–0.30	[194]
PAL‐Lys‐Lys‐NH2	Fluconazole	*C. glabrata 4812, 4849*	0.30–0.40	[194]
PAL‐Lys‐Lys‐NH2	Fluconazole	*C. guilliermondii 195, 4783*	0.25–0.40	[194]
Dodecapeptide	Fluconazole	*C. albicans*, *C. neoformans*, *A. fumigatus*	0.20–0.50	[193]
RWWRWFIFH	Fluconazole	*Candida* species and *C. neoformans*	N/A	[192]
Lactoferrin‐derived peptide hLF(1–11)	Fluconazole	*Candida* species	*P* < 0.05	[191]
Lactoferricin B	Fluconazole	*C. albicans* TIMM3315	0.13	[207]
Lactoferricin B	Fluconazole	*C. albicans* TIMM3317	0.13	[207]

^a)^ All sequences are listed in the Supporting Information.

**Table 9:** In vitro antimicrobial synergy between AMP and caspofungin

AMP^a)^	Antifungal	Organism	FICI	Refs.
LL‐37	Caspofungin	*C. auris* various strains	0.13–0.26	[206]
rHsAFP1	Caspofungin	*C. albicans*	0.21–0.50	[214]
HsLin06 not included in literature	Caspofungin	*C. albicans*	0.28–0.31	[214]
Tyrocidines A	Caspofungin	*C. albicans*	0.10–0.18	[213]
Tyrocidines B	Caspofungin	*C. albicans*	0.03–0.35	[213]
Tyrocidines C	Caspofungin	*C. albicans*	0.12–0.35	[213]
hMUC7–12, DsS3(1–16), hLF(1–11), and colistin	Caspofungin	*C. albicans*	0.094–0.164	[212]
Hepcidin‐20	Caspofungin	*C. glabrata*	N/A	[195]
PAL‐Lys‐Lys‐NH2	Caspofungin	*C. parapsilosis*	0.25–0.50	[194]
PAL‐Lys‐Lys‐NH2	Caspofungin	*C. albicans ATCC90029, 4896, 4890*	0.12–0.50	[194]
PAL‐Lys‐Lys‐NH2	Caspofungin	*C. tropicalis* ATCC750, 4795, 4867	0.25–0.40	[194]
PAL‐Lys‐Lys‐NH2	Caspofungin	*C. krusei ATCC6258, 4684*	0.30–0.50	[194]
PAL‐Lys‐Lys‐NH2	Caspofungin	*C. glabrata 4812, 4849*	0.30–0.50	[194]

^a)^ All sequences are listed in the Supporting Information.

**Table 10:** In vitro antimicrobial synergy between AMP and amphotericin B

AMP^a)^	Antifungal	Organism	FICI	Refs.
LL‐37	Amphotericin B	*C. auris* various strains	0.13–0.31	[206]
MSI‐78	Amphotericin B	*F. solani*	0.156–0.500	[226]
h‐Lf1–11	Amphotericin B	*F. solani*	0.140–0.313	[226]
Cecropin	Amphotericin B	*F. solani*	0.140–0.312	[226]
Dq‐3162 and Dq‐1503	Amphotericin B	*C. albicans* *C. tropicalis* *C. krusei* *C. parapsilosis* *C. albicans CA1*	0.3125 0.3125 0.3125 0.375 0.5	[225]
Dq‐2562	Amphotericin B	*C. albicans* *C. tropicalis* *C. krusei* *C. parapsilosis* *C. albicans CA1*	0.3125 0.3125 0.3125 0.3125 0.5	[225]
Dq‐1319	Amphotericin B	*C. tropicalis*	0.375	[225]
Lactoferrin‐derived peptide	Amphotericin B	*C. albicans, C. glabrata* *C. neoformans* *C. deuterogattii*	0.125–0.250	[224]
DS6	Amphotericin B	*C. tropicalis ATCC 13803*	0.37	[204]
DS6	Amphotericin B	Clinical isolates of *C. tropicalis*	0.5	[204]
rHsAFP1	Amphotericin B	*C. albicans*	0.44–0.47	[214]
Bacillomycin D	Amphotericin B	Candida species	0.24–0.50	[223]
Tyrocidines A	Amphotericin B	*C. albicans*	0.23–0.41	[213]
Tyrocidines B	Amphotericin B	*C. albicans*	0.14–0.42	[213]
Tyrocidines C	Amphotericin B	*C. albicans*	0.28–0.35	[213]
KU1, KU2, KU3, and KU4	Amphotericin B	*C. albicans* SC5314, ATCC 90028	0.31–0.50	[197]
Upn‐lys4	Amphotericin B	*C. albicans* SC5314, ATCC 90028	0.35–0.50	[197]
Upn‐lys5	Amphotericin B	*C. albicans* SC5314, ATCC 90028	0.30–0.50	[197]
Upn‐lys6	Amphotericin B	*C. albicans* SC5314	0.33	[197]
PAL‐Lys‐Lys‐NH2	Amphotericin B	*C. parapsilosis*	0.50	[194]
PAL‐Lys‐Lys‐NH2	Amphotericin B	*C. albicans* 4890	0.30	[194]
PAL‐Lys‐Lys‐NH2	Amphotericin B	*C. tropicalis* ATCC750, 4795	0.18–0.50	[194]
PAL‐Lys‐Lys‐NH2	Amphotericin B	*C. krusei* 4684, 4153	0.20–0.30	[194]
PAL‐Lys‐Lys‐NH2	Amphotericin B	*C. glabrata 4812, 4849*	0.30–0.50	[194]
PAL‐Lys‐Lys‐NH2	Amphotericin B	*C. guilliermondii 195, 4783*	0.12–0.50	[194]
Hepcidin‐20	Amphotericin B	*C. glabrata*	0.5	[195]
Dodecapeptide	Amphotericin B	*C. albicans, C. neoformans*, *A. fumigatus*	0.30–0.50	[193]
pep2, Hst5, and HNP1	Amphotericin B	*C. albicans*	N/A	[221]
RWWRWFIFH	Amphotericin B	*Candida* species and *C. neoformans*	N/A	[192]
Histatin 5, Dhvar4, Dhvar5, magainin 2, and PGLa	Amphotericin B	*Candida* spp.	0.11–0.44	[227]
Dhvar5 and magainin 2	Amphotericin B	*C. neoformans*	0.13–0.41	[227]
Dhvar5 and magainin 2	Amphotericin B	*A. fumigatus*	0.28–0.41	[227]

^a)^ All sequences are listed in the Supporting Information.

#### 6.1.3 Defensins

Defensins are a major family of AMPs. They are amphipathic (2–5 kD; 29–34 amino acid) Arg‐rich peptides that contain six invariant Cys residues that form three disulfide bonds, and they can be classified as *α*‐defensins or *β*‐defensins.^[^ ^25^ ^]^ Abnormal expression of defensins has been linked to infectious diseases.^[^ ^96^ ^]^ Although the primary function of defensins appears to be antimicrobial, they may also participate in inflammation, tissue injury, and repair.^[^ ^97^ ^]^

**Figure 8:**
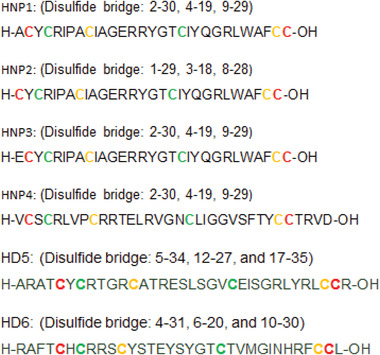
Representative *α*‐defensins.

*α*‐Defensins play key roles in the innate host defense against bacterial, fungal, and viral pathogens.^[^ ^1^, ^14^, ^98^ ^]^ Four *α*‐defensins peptides were isolated from human neutrophils and are known as human neutrophil peptides (HNP 1–4) (**Figure** **8**).^[^ ^51^ ^]^ Azurophilic protein of human neutrophils comprises HNP‐1, HNP‐3, and a low concentration of HNP‐4, whereas HNP‐1 to ‐3 is also found in B cells and natural killer cells.^[^ ^99^ ^]^ Human defensin‐5 (HD‐5) and human defensin‐6 (HD‐6) (Figure 8) are stored in specialized epithelial cells in the intestine called Paneth cells.^[^ ^100^ ^]^ HNP1–3 have been studied extensively since they are easily purified from leukocytes. However, HNP‐4 and HD‐5 and ‐6 are recovered in very small amounts and hence less is known about their properties. HD‐5 is potently bactericidal, mostly against Gram‐positive strains. By contrast, HD‐6 displays weak to no bacterial killing. However, it was recently shown to trap bacteria by forming fibril‐like structures, termed nanonets, thus contributing to intestinal homeostasis.^[^ ^101^ ^]^

*β*‐Defensins are to some extent larger than *α*‐defensins and although there are few primary homology sequences between them, their tertiary structures are very similar due to the presence of disulfide bonds. *β*‐defensins comprise HBD‐1, HBD‐2, HBD‐3, and HBD‐4 (see sequences in the Supporting Information).^[^ ^99^, ^102^ ^]^

Oral bacteria, including *Streptococcus mutans*, a primary etiological agent of dental caries, differ in sensitivity to AMPs like *α*‐defensins (HNP‐1–3), *β*‐defensins (HBD‐2–3), and human cathelicidins LL‐37. However, most combinations of these AMPs have a synergistic effect and the antimicrobial activity of each peptide against the resistant *S. mutans* is increased.^[^ ^103^ ^]^ The human cathelicidins LL‐37 and HBD‐2 combination shows synergistic antimicrobial activity by effectively killing *S*. *aureus*.^[^ ^104^ ^]^ It has been reported that HNP‐1 and guinea pig defensins (GNCPs) synergized with human cathelicidins (CAP18/LL‐37) and guinea pig cathelicidins (CAP11) to enhance antibacterial activity against *E. coli* and *S. aureus*.^[^ ^105^ ^]^ Rabbit granulocytes contain six (NP‐1, NP‐2, NP‐3a, NP‐3b, NP‐4, and NP‐5) Cys‐rich AMPs that are structurally homologous to human defensin.^[^ ^106^ ^]^ NP‐1 and NP‐2 display the greatest antibacterial,^[^ ^106b^ ^]^ antifungal,^[^ ^106c^ ^]^ and antiviral activity. NP‐1, NP‐2, and NP‐3a are also highly effective against *Candida albicans*. NP‐5 is an abundant peptide in rabbit neutrophils.^[^ ^106b^ ^]^ However, it does not have candidacidal activity. The combination of NP‐5 with low concentrations of NP‐1, NP‐2, and NP‐3a yields a synergistic effect against *Candida* species.

#### 6.1.4 Cathelicidins

Cathelicidins are a family of AMPs derived from proteins. They have a highly conserved signal sequence and a proregion that is highly homologous to cathelin, a cathepsin L inhibitor. However, the cathelicidin C‐terminal domain shows substantial heterogeneity. Cathelicidins are found mainly in cows (BMAP‐27, indolicidin, and bactenecin), pigs (protegrins), mice (CRAMP), rabbits (CAP18), and humans (hCAP‐18/LL‐37). This evolutionary conservation suggests that these AMPs play an important role in innate immunity.^[^ ^37b^ ^]^ Cathelicidin LL‐37 is a potent immunomodulator^[^ ^114^ ^]^ and it also has the following functions: it is a chemoattractant for human monocytes, T cells,^[^ ^115^ ^]^ and mast cells;^[^ ^116^ ^]^ it is a potent antiendotoxic agent;^[^ ^117^ ^]^ and it induces chemokine production.^[^ ^118^ ^]^

Pro‐rich AMPs act synergistically with cathelicidin against a variety of Gram‐negative and Gram‐positive bacterial strains.^[^ ^119^ ^]^ When combined, the synthetic AMPs L‐Ser‐Cecropin 6 and L‐Ser‐Defensin 4, identified in the transcriptome of *Lucilia* sericata, act synergistically against *E*. *coli* and *M*. *luteus*.^[^ ^120^ ^]^

The combination of indolicidin and cathelicidin LL‐37 at low concentrations acts synergistically to suppress bacterial LPS‐induced production of TNF‐*α*.^[^ ^118b^ ^]^ When used in combination with indolicin, human cathelicidin LL‐37, or bactenecin, the mammalian AMP protegrin 1 has a synergistic effect against *P*. *aeruginosa* and *E*. *coli*. The same effect against *E*. *coli* was reported when indolicin was used in combination with bactenecin.^[^ ^121^ ^]^ In addition, protegrin 1 with cathelicidin LL‐37, bactenecin with human cathelicidin LL‐37, and protegrin 1 with bactenecin combinations show synergy against *E*. *faecalis*. That study revealed that peptides from different origins can work cooperatively and give a synergistic effect.^[^ ^121^ ^]^

Human *α*‐defensin HNP‐1 and GNCPs synergized with human cathelicidin CAP18/LL‐37 and guinea pig cathelicidin CAP11 to enhance antibacterial activity against *E*. *coli* and *S*. *aureus* in the presence of 150 mm NaCl and to potentiate the outer and inner membrane permeabilization of *E. coli* ML‐35p.^[^ ^105^ ^]^ Recently, the synergistic effect of cathelicidin LL‐37 with lugdunin or gallidermin against MRSA has been reported.^[^ ^122^ ^]^

#### 6.1.5 Bacteriocins

Bacteriocins are ribosomally synthesized AMPs (toxins) produced by bacteria as a means of self‐defense to inhibit or kill other related or nonrelated bacteria. Although the producing bacteria can be affected by toxicity, they are self‐protected by immunity proteins.^[^ ^126^ ^]^ Bacteriocins can be grouped into classes I, II, and III, where the first two have three and four subclasses, respectively.

**Figure 9:**
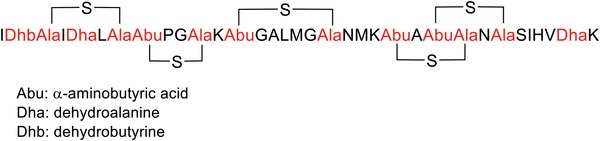
Amino acid sequence of nisin.

Class I bacteriocins (19–50 amino acids) are nonstandard amino acids, such as lanthionine, *β*‐methyllanthionine, dehydrobutyrine, dehydroalanine, and labyrinthine. These bacteriocins are a result of extensive post‐translational modification, which forms the basis of their further grouping into class Ia (lantibiotics), Ib (labyrinthopeptins), and Ic (sanctibiotics). Nisin (**Figure** **9**) is a most common class I bacteriocin.^[^ ^127^ ^]^

Class Ia comprises lantibiotics, which show antimicrobial activity at nanomolar concentrations. These AMPs are produced by Gram‐positive bacteria. A thioether containing residues termed lanthionine (Lan1) and 3‐methyllanthionine (MeLan), as well as unsaturated amino acids such as 2,3‐didehydroalanine (Dha) and 2,3‐didehydrobutyrine (Dhb) are characteristic compounds belonging to class Ia.^[^ ^128^ ^]^ Bicereucin, which is produced by *Bacillus cereus* SJ1, is a two‐component lantibiotic with unusual structural features. Unlike other previous two‐component lantibiotics mentioned, only one of the two peptides in bicereucin contains a lanthionine. The second component of bicereuin has no Cys residues; however, it does contain several D‐amino acids. Bicereucin has been reported to have synergistic antimicrobial activity against Gram‐positive strains, including MRSA and vancomycin‐resistant Enterococci (VRE).^[^ ^129^ ^]^

Lacticin 3147 (59 amino acids) is a bacteriocin isolated from *Lactococcus lactis* that contains the peptides LtnA1 (30 amino acids) and LtnA2 (29 amino acids), which act synergistically at nanomolar concentration range. This lantibiotic has been shown to work by targeting lipid II, an essential precursor of cell wall biosynthesis. LtnA1 first interacts specifically with lipid II in the outer sheet of the bacterial cytoplasmic membrane, forming a lipid II:LtnA1 complex. The complex then binds LtnA2, leading to a high‐affinity, ternary complex that subsequently inhibits cell wall biogenesis in combination with pore formation.^[^ ^130^ ^]^ The another lantiobiotic is lichenicidin VK21, which contains LchR (3249.51 Da) and Lch*β* (3019.36 Da) isolated from *Bacillus licheniformis* VK21. Each peptide has 31 amino acid residues linked by 4 intramolecular thioether bridges and the N‐terminal 2‐oxobutyryl group. These two peptides act synergistically when used together.^[^ ^131^ ^]^

Class Ib comprises carbacyclic lantibiotics containing labyrinthin and labionin. The most known example of this class is labyrinthopeptin A1 (Laby A1), which shows broad activity against human immunodeficiency virus (HIV) and herpes simplex virus. In combination with anti (retro) viral drugs, such as tenofovir, acyclovir, saquinavir, raltegravir, and enfuvirtide, Laby A1 shows additive synergistic effects.^[^ ^132^ ^]^

Class Ic is formed by sactibiotics, which are a relatively newly named subgroup of bacteriocins that includes subtilosin A, thurincin H, propionicin F, and thuricin CD.^[^ ^133^ ^]^ Thuricin CD is a binary bacteriocin produced by *Bacillus thuringiensis* DPC 643. The two components of thuricin CD, namely, Trn‐*α* and Trn‐*β*, show highly potent synergy against the human pathogen *Clostridium difficile*.^[^ ^134^ ^]^

Class IIa is characterized by antilisterial activity and a high degree of sequence homology at the N‐terminus, including the YGNGV consensus sequence and a disulfide bridge.^[^ ^18^, ^135^ ^]^ This class includes Pediocin PA‐1, sakacins A and P, and leucocin A. Pediocin‐like bacteriocins are the most abundant peptides in class IIa. The combination of Pediocin PA‐1 and nisin or polymyxin E against *L. monocytogenes* was reported to produce a synergistic effect, with an FIC of 0.28 and 0.19, respectively, and the MICs of these peptides increased from 10–20 to 0.47–2.5 µg mL^−1^.^[^ ^136^ ^]^

Class IIb bacteriocins contain two peptides that can be synergistic when delivered in equal amount for optimal antimicrobial activity.^[^ ^137^ ^]^ They belong to the huge group of small, cationic, heated‐stable non‐lantibiotics, generally produced by *Lantobacillus plantarum*.^[^ ^138^ ^]^ The most common examples are Plantaricin E and F (PlnE and PlnF), which have antimicrobial activity against *S. citreus* and *S. aureus*, the former showing the most activity. Furthermore, the combination of PlnE and PlnF (PlnEF) produces greater activity than each peptide alone.^[^ ^139^ ^]^ The same observation has been made for PlnJK. In this regard, the MICs of the bacteriocins PlnJ, PlnK, and combined PlnJK against *Lactobacillus plantarum* ZFM811 were 6.4, 12.8, and 0.4 µm, respectively. These findings thus indicate that PlnJK has a lower MIC and hence stronger antimicrobial activity than the other two bacteriocins individually, thus indicating the synergistic effect of the two peptides.^[^ ^140^ ^]^ The synergistic mechanism of PlnJK is thought to be due to the formation of an amphiphilic *α*‐helical structure in a membrane‐mimicking environment and permeabilized bacterial membranes in a micromolar concentration.^[^ ^141^ ^]^ The mechanism underlying the action of PlnEF has been revealed in *L. plantarum 965* cells pore former in the cytoplasmic membranes of the target cells.^[^ ^142^ ^]^ The bactericidal activity of PlnEF involves significant membrane disruption, which eventually leads to cell death.^[^ ^139^ ^]^

Most bacteriocins are originally produced with an N‐terminal extension called a leader peptide.^[^ ^143^ ^]^ However, some, such as Enterocins 7A and 7B isolated from *Enterococcus faecalis* 710C, are produced without. Leaderless bacteriocins are of particular interest due to their relatively broad spectrum of activity, inhibiting important foodborne pathogens such as *Clostridium* spp. and *Listeria monocytogenes*.^[^ ^144^ ^]^ These bacteriocins are also active against VRE and MRSA, which are common in nosocomial infections.

Class IIc, known as circular bacteriocins, is examplified by enterocin, a type of bacteriocin synthesized by lactic acid bacteria, Enterococcus. *Enterococcus faecium* L50 produces enterocin L50A and L50B (EntL50A and EntL50B) and shows a predominant antimicrobial spectrum against major beer‐spoilage lactic acid bacteria (i.e., *Lactobacillus brevis* and *Pediococcus damnosus*). Bacteriocin assays using in vitro‐synthesized EntL50A and EntL50B showed that each individual peptide exerts antibacterial activity, the former being the most active. However, upon combination, these two peptides have a synergistic effect.^[^ ^145^ ^]^

Class IId comprises unmodified, linear, leaderless, non‐pediocin‐like bacteriocins known as bactofencin A and LsbB and these act mainly on Gram‐positive bacteria. However, no combined use has been reported.^[^ ^127^ ^]^

Class III bacteriocins are large heat‐liable peptides with activity against both Gram‐positive and Gram‐negative bacteria. The most widely known members of this class are helveticin M, helveticin J, and enterolysin A.^[^ ^127^ ^]^

#### 6.1.6 Other Peptide–Peptide Synergism

Bombinins are peptides secreted from the skin of the *Bombina* frog species. BHL‐bombinin, bombinin HL, and bombinin HD were found to adopt an amphipathic *α*‐helical conformation in a membrane‐mimetic environment—a key feature that allows AMPs to exert antimicrobial activity.^[^ ^149^ ^]^ The interaction of BHL‐bombinin with either bombinin HL or bombinin HD has been described to show synergistic inhibition of *S. aureus* (FICI: 0.375).^[^ ^150^ ^]^ Bombinins have also shown synergistic antimicrobial activity when used in combination with feleucin‐BV1.^[^ ^151^ ^]^

Protonectin (PTN) and Protonectin 1–6 (PTN_1–6_) are peptides found in the venom of *Agelaia* pallipes *pallipes*,^[^ ^152^ ^]^ and they have antibacterial, antifungal, and anticancer activities.^[^ ^153^ ^]^ A 1:1 mixture of these two peptides has positive synergistic antimicrobial effects, attributed to the formation of a heterodimer, as hypothesized based on MSESI data.^[^ ^154^ ^]^

With 34 amino acids containing 10 Pro residues, abaecin, found in *Apis mellifera*, is one of the largest Pro‐rich antimicrobial peptides.^[^ ^36^ ^]^ Abaecin is synergistically enhanced by the presence of hymenoptaecin, a pore‐forming AMP. The synergy is a result of pore formation induced by hymenoptaecin, potentially causing cell leakage or lytic cell death and enabling abaecin to enter bacterial cells. Abaecin binds to Dnak, a molecular chaperon, to inhibit bacterial replication.^[^ ^155^ ^]^ Other insect AMPs, diptericins, and attacins, show synergistic effects against *P. burhodogranariea* in flies.

There are numerous reports of peptide–peptide synergism.^[^ ^120^, ^122^, ^156^ ^]^ Many studies have described antimicrobial synergies with two AMPs, while others show synergy with three. For example, the triple combination of apidaecin, pexiganan, and LL 19–27 shows strong synergism.^[^ ^157^ ^]^ The combinations of human *β*‐defensin, LL‐37, and lysozyme, which are produced on the skin, are reported to have potent synergistic effects against *S*. *aureus* and *E*. *coli*.^[^ ^158^ ^]^

### 6.2 AMP–Antibiotic Synergism

The combination of AMPs and antibiotics can produce synergistic action and, in some instances, it can overcome antibiotic resistance. Using AMPs to increase the efficacy of already authorized antibiotics seems to be a promising option to fight drug‐resistant pathogens. The human AMPs cathelicidins LL‐37 and *β*‐defensin 3 (HBD‐3) have antimicrobial synergy with the antibiotics tigecycline, moxifloxacin, piperacillin–tazobactam, and meropenem. In particular, antibiotic killing against *C. difficile* is improved when human cathelicidin LL‐37 and HBD‐3 are present.^[^ ^159^ ^]^ Ultimately, LL 17–29 establishes antimicrobial synergy with the antibiotic chloramphenicol against notably virulent bacterial strains, together with MRSA and multidrug‐resistant *P. aeruginosa*. Combining the AMPs nisin Z, pediocin, or colistin with numerous antibiotics, including penicillin, ampicillin,^[^ ^160^ ^]^ and rifampicin, is effective in overcoming antibiotic‐resistance in *P. fluorescens*. Additionally, the AMP melamine has synergistic activity when paired with ciprofloxacin, a fluoroquinolone antibiotic, against antibiotic‐resistant strains of *P. aeruginosa*. This combination may also be useful to overcome *P. aeruginosa* resistance to fluoroquinolone antibiotics.^[^ ^161^ ^]^ Synergistic combinations of AMPs with polymyxin B, erythromycin, and tetracycline have also been reported. In particular, variants of the AMP indolicidin synergize with the antibiotics polymyxin B, tobramycin,^[^ ^162^ ^]^ gentamycin, and amikacin. One of the mechanisms by which AMPs enhance antibiotic characteristics is by disrupting bacterial membranes, thereby facilitating the transport of antibiotics into the bacterial cytoplasm, wherein they can act on intracellular targets. As an example, the AMP arenicin‐1 synergistically functions with antibiotics such as ampicillin, erythromycin, and chloramphenicol to kill *S. aureus*, *S. dermis*, *P. aeruginosa*, and *E. coli*. Arenicin‐1 assists in the uptake of antibiotics into cells and inhibits bacterial growth through hydroxyl radical formation. These observations thus suggest complementary mechanisms are at play.^[^ ^163^ ^]^

### 6.3 AMP–Antifungal Synergism

#### 6.3.1 Fluconazole

Fluconazole is a bis‐triazole antifungal drug with a pharmacokinetic profile characterized by its high‐water solubility, low affinity for plasma proteins, and metabolic stability, and it is used to treat vaginal candidiasis.^[^ ^186^ ^]^ Fluconazole acts by obstructing the conversion of fungal lanosterol to ergosterol, thereby inhibiting membrane sterol synthesis and preventing fungal cell replication. It is commonly prescribed to patients with compromised immunity.^[^ ^187^ ^]^ However, many fungi have gained resistance to this fungistatic agent.^[^ ^188^ ^]^ To improve its activity, it has been used in combination with other compounds.^[^ ^189^ ^]^

The highly azole‐resistant strain TIMM3317 is resistant to the presence of fluconazole or itraconazole. However, the addition of lactoferricin B has been shown to have a synergistic effect against the growth of *Candida albicans* hyphae.^[^ ^190^ ^]^ Lactoferrin‐derived peptide hLF(1–11) is highly active against fluconazole‐resistant *C*. *albicans*, and when used in combination with fluconazole it acts synergistically against this yeast and a fluconazole‐sensitive *Candida* species. Exposure of *Candida* strains to hLF(1–11) for 5 min and then incubation with fluconazole effectively kills the yeast. However, no candidacidal activity occurs when the yeast is first incubated with fluconazole and then exposed to the peptide. This observation thus indicates that the candidacidal activity is initiated first by the peptide, while fluconazole is needed only during the effector phase.^[^ ^191^ ^]^

Fluconazole was also found to be synergistic with the peptide RWWRWFIFH against *C*. *albicans* ATCC 2091 and *C*. *albicans* ATCC 10231.^[^ ^192^ ^]^ Combined treatment with a dodecapeptide and fluconazole revealed a synergistic effect against *C*. *albicans*, *Cryptococcus neoformans*, and *Aspergillus fumigatus*.^[^ ^193^ ^]^ The short lipopeptide palmitoyl PAL‐Lys‐Lys‐NH_2_ Palmitoyl (PAL) was reported to be synergistic with fluconazole against several species of *Candida*.^[^ ^194^ ^]^ The human cationic peptide hepcidin 20 (50 µg mL^−1^) and fluconazole (64 µg mL^−1^) exert a synergistic effect, with an FICI value of 0.5 against resistant *Candida glabrata*.^[^ ^195^ ^]^ Synergy was found in 35%, 30%, and 25% of IB‐367‐fluconazole, IB367‐itraconazole, and IB‐367‐TERB interactions, respectively, against *Trichophyton* and *Microsporum* species.^[^ ^196^ ^]^

Fluconazole also showed synergy with four tryptophan‐containing peptides (KU4, KABT‐AMP, Upn‐lys6, and uperin 3.6), with improved hydrophobicity against ATCC 90028 strain.^[^ ^197^ ^]^ The synergy might be due to the increased hydrophobicity of the peptide, which is one of the crucial parameters responsible for the antimicrobial activity of AMPs.^[^ ^198^ ^]^ Increased hydrophobicity facilitates AMP‐membrane interaction and determines the extent of partition of AMPs into the cell, increasing the overall antimicrobial activity of these molecules.^[^ ^199^ ^]^ Also, cationicity conferred by the tryptophan hydrophobic residues which has a strong preference for the interfacial region of the lipid bilayers of the yeast cell membrane contributes to synergistic effect.^[^ ^200^ ^]^ The synthetic antimicrobial *β*‐peptide was used in combination either with fluconazole or ketoconazole and showed synergy by enhancing the in vitro inhibition of planktonic and biofilm *C. albicans*.^[^ ^201^ ^]^

According to a study by Taveira et al., when used in combination with fluconazole, the ChaThi thionin‐like (CaThi) peptide gives a synergistic effect against the growth of *Candida albicans*, *C*. *tropicalis*, *C*. *parapsilosis*, *C*. *pelliculosa*, *C*. *buinensis*, and *C*. *mogii* by 77.5%, 96.26%, 100.0%, 57.45%, 67.01%, and 61.05%, respectively.^[^ ^202^ ^]^ CaThi has strong candidacidal activity against six pathogenic *Candida* species, where it has nuclear intracellular target in these yeasts. It works by permeabilizing the membrane and inducing an oxidative stress response. The fluconazole and CaThi combination is synergistic because it increases growth inhibition and causes dramatic morphological changes in all the *Candida* species tested.^[^ ^202^, ^203^ ^]^ The DS6 peptide was found to be synergistic with fluconazole against *Candida* species.^[^ ^204^ ^]^ The peptide Cc‐GRP at 400 µg mL^−1^ in combination with fluconazole at 20 µg mL^−1^ was reported to inhibit the growth of the fungus *Fusarium solani*, promote the permeabilization of its membrane, and induce the production of ROS, thereby suggesting synergistic activity between the peptide and fluconazole.^[^ ^205^ ^]^ The human cathelicidin LL‐37:fluconazole combination at a 1:1 ratio results in 70% synergy against *C. auris*.^[^ ^206^ ^]^

#### 6.3.2 Caspofungin

Caspofungin acetate is a semisynthetic water‐soluble lipopeptide produced from the fermentation product of the fungus *Glarea lozoyensis*. It belongs to the echinocandin family and is a derivative of pneumocandin B_o_. Caspofungin blocks the synthesis of fungal cell walls by noncompetitive inhibition of the enzyme *β*(1,3)‐d‐glucan synthase, which is essential for *β*(1,3)‐d‐glucan synthesis, a component of the cell wall of many fungi. The *β*(1,3)‐d‐glucan chains form a solid 3D matrix that gives the cell wall its shape and mechanical strength. Inhibition of *β*(1,3)‐d‐glucan synthesis results in dual effects, both fungistatic and fungicidal.^[^ ^208^ ^]^

Caspofungin is effective against yeasts of the genus *Candida* (including isolates resistant to azoles and amphotericin B), several species of filamentous fungi, including *Aspergillus*, and certain dimorphic fungi, such as *Histoplasma*, *Blastomyces*, and *Coccidioides*.^[^ ^209^ ^]^ Recently, caspofungin has been used in combination with other antifungals and compounds.^[^ ^210^ ^]^

The combination of the echinocandins caspofungin or anidulafungin with a range of structurally diverse AMPs results in the potent synergistic killing of *Candida* spp. in vitro.^[^ ^211^ ^]^ The short lipopeptide palmitoyl PAL‐Lys‐Lys‐NH2 (PAL) was found to be synergistic with caspofungin against several species of *Candida*.^[^ ^194^ ^]^ Caspofungin has been used in combination with hepcidin‐20 peptide and shows a good synergistic effect against *C. glabrata*.^[^ ^195^ ^]^ Caspofungin in combination with each of the AMPs (hMUC7–12, DsS3(1–16), hLF(1–11)) and colistin are synergistic and candidacidal against *Candida* species in vitro.^[^ ^212^ ^]^ The tyrocidines A peptide and caspofungin combination potentiate the activity of caspofungin against *Candida albicans* biofilms.^[^ ^213^ ^]^ The plant defensin rHsAFP1 was used in combination with caspofungin and has a synergistic effect on *C*. *albicans*.^[^ ^214^ ^]^ The human cathelicidin LL‐3 peptide and caspofungin combination interaction in a 1:1 ratio results in 100% synergy against *Candida auris*.^[^ ^206^ ^]^

#### 6.3.3 Amphotericin B

Amphotericins A and B were isolated in 1959 as by‐products of the fermentation process of the soil actinomycete *Streptomyces nodosus*.^[^ ^215^ ^]^ Amphotericin B is a member of the polyene macrolide class of antibiotics. The activity of amphotericin B may be fungistatic or fungicidal (depending on drug concentration and organism susceptibility) and it is affected by pH in vitro.^[^ ^216^ ^]^ Its mechanism of action is based, at least in part, on binding to a sterol (ergosterol) present in the membrane of susceptible fungi.^[^ ^217^ ^]^ Polyenes alter membrane permeability and cause leakage of cellular components, with subsequent cell death.^[^ ^218^ ^]^ Amphotericin B can also stimulate cell proliferation and has potent immunostimulatory effects in mice.^[^ ^219^ ^]^ Amphotericin B has shown activity against various fungal species, including *Torulopsis glabrata*, *Blastomyces dermatitidis*, *Coccidioides immitis*, *Cryptococcus neoformans*, *Paracoccidioides brasiliensis*, *Histoplasma capsulatum*, and *Sporothrix* spp. However, toxicity and resistance are issues.^[^ ^220^ ^]^

Used in combination with amphotericin B, peptides like histatin 5, Dhvar4, Dhvar5, magainin 2, and PGLa all show a magnificent synergistic effect against *Aspergillus*, *Candida*, and *Cryptococcus* strains, and against an amphotericin B‐resistant *C. albicans* laboratory mutant. Amphotericin B and peptide RWWRWFIFH exert a synergistic effect against *C. albicans* ATCC 2091 and *C. albicans* ATCC 1023.^[^ ^192^ ^]^ Pep2, Hst5, and HNP1 synergistically cooperate with amphotericin B and itraconazole by promoting the efflux of ATP from Candida cells and suppressing Candida colony formation.^[^ ^221^ ^]^ When used with PAL‐Lys‐Lys‐NH2 peptide, amphotericin B shows synergy against *Cryptococcus neoformans*.^[^ ^222^ ^]^ The combination of 25 µg mL^−1^ of Hepcidin‐20 and 1 µg mL^−1^ amphotericin B against resistant *C. glabrata* BPY44 shows an enhanced fungicidal effect at low concentrations, suggesting a synergistic effect.^[^ ^195^ ^]^ The short lipopeptide palmitoyl PAL‐Lys‐Lys‐NH2 (PAL) was found to be synergistic with amphotericin B against several species of *Candida* and *Cryptococcus neoformans*.^[^ ^194^, ^222^ ^]^ In combination with amphotericin B, the three major tyrocidines (TrcA, TrcB, and TrcC) have been reported to show a synergistic effect against *Candida albicans* biofilms.^[^ ^213^ ^]^ The plant defensin rHsAFP1 in combination with amphotericin B has a synergistic effect against *C. albicans*.^[^ ^214^ ^]^

All the KU1‐KU4, Upn‐lys4‐Upn‐lys6, KABT‐AMP, and Uperin 3.6 peptides in combination with amphotericin B show synergistic effects, with FIC indices of less than 0.5 when tested against *C*. *albicans* SC5314 strain. For *C*. *albicans* ATCC 90028 strain, all the peptides exhibit synergistic effects with amphotericin B, except for Upn‐Lys6 (FICI 0.58) and uperin 3.6 (FICI 0.67).^[^ ^197^ ^]^ The lipopeptide bacillomycin D in combination with amphotericin B at their subinhibitory concentration is synergistic against pathogenic *Candida* species.^[^ ^223^ ^]^ The DS6 peptide was found to be synergistic with amphotericin B against both the clinical isolates and ATCC 13803 with FICI 0.5 and 0.37, respectively.^[^ ^204^ ^]^ Lactoferrin‐derived peptide used in combination with amphotericin B is synergistic in both *Candida* species (FICI = 0.375) and *C. neoformans* (FICI = 0.5) but indifferent in *C. deuterogattii* (FICI = 0.75).^[^ ^224^ ^]^

The combination of synthetic pilosulin‐ (Dq‐2562, Dq‐1503, and Dq‐1319) and ponericin‐like (Dq‐3162) peptides and conventional antimycotic drugs, including amphotericin B, displays a synergistic reduction in the MIC values of individual peptides and drugs, in which underlying mechanism of action involves membrane disruption of *Candida* spp.^[^ ^225^ ^]^ AMPs such as MSI‐78, h‐Lf1‐11, and cecropin B combined with amphotericin B and voriconazole antifungals has a significant synergistic effect against Fusarium species.^[^ ^226^ ^]^ The human cathelicidin LL‐37:amphotericin B combination at a 1:1 ratio results in 100% synergy against *Candida auris*.^[^ ^206^ ^]^

#### 6.3.4 Clotrimazole

Clotrimazole is a synthetic broad‐spectrum antifungal drug primarily used for the treatment of *Candida albicans* and other fungal infections.^[^ ^228^ ^]^ Clotrimazole is commonly used as a topical treatment for tinea pedis (athlete's foot), as well as vulvovaginal candidiasis and oropharyngeal candidiasis.^[^ ^229^ ^]^ By targeting ergosterol biosynthesis, it exhibits fungistatic antimycotic activity, thereby inhibiting fungal growth. Clotrimazole inhibits the microsomal cytochrome P450 (CYP450)‐dependent event 14‐*α*‐lanosterol demethylation, a key step in fungal ergosterol biosynthesis by fungi.^[^ ^230^ ^]^ The concentration of lactoferricin B required to inhibit Candida growth is reduced in the presence of relatively low concentrations of clotrimazole, suggesting a synergistic effect between the two antifungals.^[^ ^231^ ^]^

#### 6.3.5 Flucytosine

Flucytosine is a synthetic antimycotic compound, first prepared in 1957. Flucytosine (5‐FC) action takes effect when it encounters the fungus and is taken up by an enzyme known as cytosine permease. Once the drug enters the fungus, it is converted to its active form 5‐fluorouracil (5‐FU) by the enzyme cytosine deaminase within the cell. 5‐Fluorouracil incorporates itself into the RNA strand by competing with uracil, disrupting RNA synthesis, and impairing protein synthesis within the fungus. 5‐Fluorouracil also further inhibits DNA synthesis through its conversion into fluoro‐deoxyuridylic acid and inhibits thymidylate synthase, which causes DNA damage within fungal cells.^[^ ^232^ ^]^

Susceptible fungi contain cytosine deaminase, an enzyme considered absent from human cells. This enzyme rapidly converts flucytosine to the antimetabolite, 5‐fluorouracil. Flucytosine is most active against yeast, including *Candida* spp., *Torulopsis*, *Cryptococcus*, dematiaceous fungi causing chromomycosis, and *Aspergillus* spp.^[^ ^233^ ^]^ Flucytosine is scarcely used alone because of the development of resistance.^[^ ^234^ ^]^ The combination of peptide RWWRWFIFH with flucytosine was found to be synergistic on three occasions against *C. albicans*, where a significant decrease in total viable count was observed.^[^ ^192^ ^]^ Flucytosine has been used in combination with a peptide hepcidin‐20 and shows a good synergy between the two compounds (**Table** **11**).^[^ ^193^ ^]^

**Table 11:** In vitro antimicrobial synergy between AMPs and various antifungal drugs

AMP^a)^	Antifungal	Organism	FICI	Refs.
MSI‐78 and cecropin	Voriconazole	*F. solani*	0.141–0.375	[226]
hLF1–11	Voriconazole	*F. solani*	0.078–0.375	[226]
Cecropin	Voriconazole	*F. solani*	0.140–0.312	[226]
*β*‐peptide	Ketoconazole	*C. albicans*	0.25	[201]
IB‐367	Itraconazole	*T. mentagrophytes*, *T. rubrum*, and *M. canis*	N/A	[196]
IB‐367	Terbinafine	*T. mentagrophytes*, *T. rubrum*, and *M. canis*	N/A	[196]
Tachyplesin III	Terbinafine	*T. mentagrophytes* 100	0.12	[196]
Tachyplesin III	Terbinafine	*T. rubrum* 131 and 136	0.09–0.18	[196]
Tachyplesin III	Terbinafine	*M. canis* 132 and 133	0.18–0.50	[196]
Tachyplesin III	Terbinafine	*M. gypseum* 144	0.50	[196]
Dodecapeptide	Flucytosine	*C. albicans*, *C.neoformans*, *A. fumigatus*	0.10–0.50	[193]
RWWRWFIFH	Flucytosine	*Candida* species and *C. neoformans*	N/A	[192]
RWWRWFIFH	Ketoconazole	*Candida* species and *C. neoformans*	N/A	[192]
Lactoferricin B	Itraconazole	*C. albicans* TIMM3315, *C. albicans* TIMM3317	0.13 for both	[190]
Lactoferricin B	Clothrimazole	*C. albicans*	N/A	[190]

^a)^ All sequences are listed in the Supporting Information.

#### 6.3.6 Other Antifungal Agents

Many other peptide–antifungal agent combinations have shown synergy. The ketoconazole antifungal agent and peptide RWWRWFIFH are synergistic against *C. albicans*.^[^ ^192^ ^]^ Ketoconazole is also synergistic with a *β*‐peptide against *C. albicans* SC5314 strain, the synergism is speculated to be due to the combination of the different mechanistic routes of the agents: the *β*‐peptide has a membrane‐permeating nature plus the ergosterol biosynthesis inhibition caused by the ketoconazole.^[^ ^201^ ^]^ Peptides and peptide‐like compounds have been reported to generate a hypersynergistic antifungal effect with rapamycin on both azole‐resistant and sensitive clinical *C. albicans* isolates.^[^ ^235^ ^]^ In combination with tachyplesin III, the terbinafine antifungal agent shows a synergistic effect.^[^ ^236^ ^]^

## 7 Conclusion and Future Perspectives

Infectious diseases are one of the main causes of the death, mostly of them due to antimicrobial resistance. The use of AMPs is seen as one of the best strategies to fight against these diseases. The combination of AMPs with other conventional antibiotics has been demonstrated in vitro a great potential for reversing the antimicrobial resistance. Although this paradigm has not yet demonstrated in clinic, the use of combination drugs is well accepted by the medicinal agencies. Thus, in the period 2016–2022, 19 combination drugs, where two or more active pharmaceutical ingredients (APIs) are present, have been authorized by the US Food and Drug Administration (FDA) [343, REF]. This combination approach mainly started in the retroviral field to treat hepatitis and HIV infection. Then, several antibiotic combination drugs have been approved. Recarbrio (formed by imipenem, cilastatin, and relebactam) and Vabomere (which combines meropenem and vaborbactam) to treat complicated urinary tract infections; and in 2022, VoqueznaTriple Pak with vonoprazan, amoxicillin, and clarithromycin was authorized to treat *Helicobacter pylori*.

These combination treatments are usually accompanied by synergistic effects, allowing the use of lower doses of drugs compared to monodrug therapy, thereby limiting the adverse effects of medications. Enhancing antibiotic effectiveness and the lower doses required may be highly beneficial in an era of ever‐increasing bacterial resistance to conventional antimicrobial therapy. However, it is crucial to remember that combined therapies' synergistic effects may also increase the toxic effect of each drug compared to monotherapy. Therefore, extreme attention should be devoted to studies on drug combinations. Moreover, such combinations can broaden the spectrum of antimicrobial activity, minimizing the selection of resistant microorganisms and increasing safety and tolerance by using lower drug doses. Furthermore, this strategy can also fuel the repurposing of conventional antimicrobial agents to which various microorganisms have developed resistance or that have been showed to be too toxic and, therefore, their development has been previously abandoned.

Overall, it is envisaged that in the near future, AMPs will be part of preclinical and clinical studies based on the use of combination drugs, and therefore some of these combinations will reach the market. However, more basic multidisciplinary research should first be developed for a complete understanding of the mode of action of the AMPs to combat microorganisms and their interaction with other AMPs, but also with the so‐called small molecule antibiotics, for obtaining synergies. So far, most studies carried out and discussed in this review are in vitro. In this regard, more work should be done using animal models for a complete understanding of the scope and limitations of this strategy.

Another strategy to be investigated is the use of AMPs forming part of antimicrobial polymer combinations. AMPs could have the potential of being part of the polymer or as a component of the combination by itself. For instance, as polymers, peptides can form part of dendrimers as branching or decorating moieties. It is believed that antimicrobial polymers could be an effective way of tackling antibiotic resistance.^[^ ^237^ ^]^

Peptides can also be exploited as bacteria penetrating peptides. This will be similar to cell penetrating peptides (CPPs), which have been demonstrated to be essential in introducing conventional drugs inside the cells. So far, CPPs, due to their cationic structure and detent antimicrobial properties, are being used in research to assist antibiotics in penetrating the bacteria. The development of more specific cell‐penetrating peptides for antibiotics should enhance the effectiveness of this strategy.

Another approach that can find its roots in the use of combination drugs is building in the same molecule the conjugation of two moieties, the antibiotic, and the peptide, through a linker. This combination should render single chemical entities, peptide drug conjugates or peptide antibiotic conjugates, similar to the antibody drug conjugates.^[^ ^238^ ^]^

As final remark, exploiting the availability of AMPs when are used in combination with other antimicrobial molecules is called to be one of the best strategies to enhance effectiveness of the antibiotics anminimizes their resistance created by the large amounts of antibiotic used. However, combination therapy poses more complexity to the already complex world of drug discovery. Formulation and dosing, administration method, and pharmacokinetics of each component should be carefully analyzed to avoid negative and, in some cases, fatal outcomes.^[^ ^238^ ^]^

## Conflict of Interest

The authors declare no conflict of interest.

## Supporting information



Supporting Information
