# Supplementary material for: Antimicrobial Peptide Synergies for Fighting Infectious Diseases
Source: Adv Sci (Weinh). 2023 Jul 5;10(26):2300472. doi: 10.1002/advs.202300472 (PMC10502873; doi:10.1002/advs.202300472)
Supplement: Supplementary file 1 — Supporting Information [file ADVS-10-2300472-s001.pdf]

ADVANCED SCIENCE
Open Access

Supporting Information

for *Adv. Sci.*, DOI 10.1002/advs.202300472

Antimicrobial Peptide Synergies for Fighting Infectious Diseases

Jessica T. Mhlongo, Ayman Y. Waddad, Fernando Albericio* and Beatriz G. de la Torre**

Supplementary Information

Antimicrobial Peptide Synergy for Fighting Infectious Diseases

Jessica T. Mhlongo,[1,2] Ayman Y. Waddad,[2,*] Fernando Albericio,[2,3,*] Beatriz G. de la Torre[1,*]

[1] KwaZulu-Natal Research Innovation and Sequencing Platform (KRISP), School of Laboratory Medicine and Medical Sciences, College of Health Sciences, University of KwaZulu-Natal, Durban 4041, South Africa.

[2] Peptide Science Laboratory, School of Chemistry and Physics, University of KwaZulu-Natal, Westville, Durban 4000, South Africa.

[3] CIBER-BBN, Networking Centre on Bioengineering, Biomaterials and Nanomedicine, and Department of Organic Chemistry, University of Barcelona, 08028 Barcelona, Spain.

AMP PEPTIDE SEQUENCES

(in the order that they appeared in the main text)

Magainin 2:

H-Gly-Ile-Gly-Lys-Phe-Leu-His-Ser-Ala-Lys-Lys-Phe-Gly-Lys-Ala-Phe-Val-Gly-Glu-Ile-Met-Asn-Ser-OH [1]

Magainin 2a:

H-Gly-Ile-Gly-Lys-Phe-Leu-His-Ser-Ala-Lys-Lys-Phe-Gly-Lys-Ala-Phe-Val-Gly-Glu-Ile-Met-Asn-Ser-NH_2 [2]

PGLa:

H-Gly-Met-Ala-Ser-Lys-Ala-Gly-Ala-Ile-Ala-Gly-Lys-Ile-Ala-Lys-Val-Ala-**Leu-**Lys-Ala-Leu-NH_2 [1]

L18W-PGLa:

H-Gly-Met-Ala-Ser-Lys-Ala-Gly-Ala-Ile-Ala-Gly-Lys-Ile-Ala-Lys-Val-Ala-**Trp**-Lys-Ala-Leu-NH_2 [2]

Tachyplesin I:

H-Lys-Trp-Cys-Phe-Arg-Val-Cys-Tyr-Arg-Gly-Ile-Cys-Tyr-Arg-Arg-Cys-Arg-NH_2 [3]

Temporin A: (Also named Temporin 1Ta)*****

H-Phe-Leu-Pro-Leu-Ile-Gly-Arg-Val-Leu-Ser-Gly-Ile-Leu-NH_2 [3]

Temporin B: (Also named Temporin 1Tb)

H-Leu-Leu-Pro-Ile-Val-Gly-Asn-Leu-Leu-Lys-Ser-Leu-Leu-NH_2 [4]

Temporin L: (Also named Temporin 1Tl)

H-Phe-Val-Gln-Trp-Phe-Ser-Lys-Phe-Leu-Gly-Arg-Ile-Leu-NH_2 [4]

Temporin TB-YK:

H-**Lys-Lys-Tyr**-Leu-Leu-Pro-Ile-Val-Gly-Asn-Leu-Leu-Lys-Ser-Leu-Leu-NH_2 [4]

*****Temporins from A to L now re-named temporins 1Ta–1Tl, according to the new nomenclature proposed by reference [5]

RJ1-C: (C-terminal modified)

H-Pro-Phe-Lys-Ile-Asp-Ile-His-Leu-Gly-Gly-Tyr-NH_2

Citropin 1.1:

H-Gly-Leu-Phe-Asp-Val-Ile-Lys-Lys-Val-Ala-Ser-Val-Ile-Gly-Gly-Leu-NH_2 [6]

CA(1–7)M(2–9)NH_2:

H-Lys-Trp-Lys-Leu-Phe-Lys-Lys-Ile-Gly-Ala-Val-Leu-Lys-Val-Leu-NH_2 [6]

Pal-K-G-K-NH2:

Palmitoyl-Lys-Gly-Lys-NH_2 [6]

HNP1:

H-Ala-Cys (&^{1})-Tyr-Cys(&^{2})-Arg-Ile-Pro-Ala-Cys(&)^3-Ile-Ala-Gly-Glu-Arg-Arg-Tyr-Gly-Thr- Cys(&^{2})-Ile-Tyr-Gln-Gly-Arg-Leu-Trp-Ala-Phe-Cys(&^{3})-Cys(&^{1})-OH [7, 8]

HNP2:

H-Cys(&^{1})-Tyr-Cys(&^{2})-Arg-Ile-Pro-Ala-Cys(&^{3})-Ile-Ala-Gly-Glu-Arg-Arg-Tyr-Gly-Thr-Cys(&^{2})-Ile-Tyr-Gln-Gly-Arg-Leu-Trp-Ala-Phe-Cys(&^{3})-Cys(&^{1})-OH [7, 8]

HNP3:

H-Asp-Cys(&^{1})-Tyr-Cys(&^{2})-Arg-Ile-Pro-Ala-Cys(&^{3})-Ile-Ala-Gly-Glu-Arg-Arg-Tyr-Gly-Thr-Cys(&^{2})-Ile-Tyr-Gln-Gly-Arg-Leu-Trp-Ala-Phe-Cys(&^{3})-Cys(&^{1})-OH [7, 8]

HNP4:

H-Val-Cys(&^{1})-Ser-Cys(&^{2})-Arg-Leu-Val-Phe-Cys(&^{3})-Arg-Arg-Thr-Glu-Leu-Arg-Val-Gly-Asn-Cys(&^{2})-Leu-Ile-Gly-Gly-Val-Ser-Phe-Thr-Tyr-Cys(&^{3})-Cys(&^{1})-Thr-Arg -Val-Asp-OH[7, 8]

HD5:

H-Ala-Arg-Ala-Thr-Cys(&^{1})-Tyr-Cys(&^{2})-Arg-Thr-Gly-Arg-Cys(&^{3})-Ala-Thr-Arg-Glu-Ser-Leu-Ser-Gly-Val-Cys(&^{2})-Glu-Ile-Ser-Gly-Arg-Leu-Tyr-Arg-Leu-Cys(&^{3})-Cys(&^{1})-Arg-OH[8]

HD6:

H-Arg-Ala-Phe-Thr-Cys(&^{1})-His-Cys(&^{2})-Arg-Arg-Ser-Cys(&^{3})-Tyr-Ser-Thr-Glu-Tyr-Ser-Tyr-Gly-Thr-Cys(&^{2})-Thr-Val-Met-Gly-Ile-Asn-His-Arg-Phe-Cys(&^{3})-Cys(&^{1})-Leu[8]

hBD-1

H-DHYNC(&^{1})VSSGGQC(&^{2})LYSAC(&^{3})PIFTKIQGTC(&^{2})YRGKAKC(&^{3})C(&^{1})K-OH [8]

hBD-2

H-DPVTC(&^{1})LKSGAIC(&^{2})HPVFC(&^{3})PRRYKQIGTC(&^{2})GLPGTKC(&^{3})C(&^{1})KKP-OH [8]

hBD-3

H-QKYYC(&^{1})RVRGGRC(&^{2})AVLSC(&^{3})LPKEEQIGKC(&^{2})STRGRKC(&^{3})C(&^{1})RRKK-OH [8]

hBD-4

H-ELDRIC(&^{1})GYGTARC(&^{2})RKKC(&^{3})RSQEYRIGRC(&^{2})PNTY C(&^{3})C(&^{1})LRK-OH [8]

Cg-Defm

H-GFGC(&^{1})PGNQLKC(&^{2})NNHC(&^{3})KSISC(&^{4})RAGYC(&^{1})DAATLWLRC(&^{2})TC(&^{3})TDC(&^{4})NGKK-OH [9, 10]

Cg-Defh1

H-GFGC(&^{1})PGDQYEC(&^{2})NRHC(&^{3})RSIGC(&^{4})RAGYC(&^{1})DAVTLWLRC(&^{2})TC(&^{3})TGC(&^{4})SGKK-OH [9, 10]

Cg-Defh2

H-GFGC(&^{1})PRDQYKC(&^{2})NSHC(&^{3})QSIGC(&^{4})RAGYC(&^{1})DAVTLWLRC(&^{2})TC(&^{3})TDC(&^{4})NGKK-OH [9, 10]

Cg-IgPrp

H-Asp-Thr-Gly-Pro-Ile-Arg-Arg-Pro-Lys-Pro-Arg-Pro-Arg-Pro-Arg-Pro-Glu-Gly[10]

Cg-PRP22–36

H-Gly-Pro-Ile-Arg-Arg-Pro-Lys-Pro-Arg-Pro-Arg-Pro-Arg-Pro-Glu-NH_2 [11]

Cg-PRP26–36

H-Arg-Pro-Lys-Pro-Arg-Pro-Arg-Pro-Arg-Pro-Glu-NH_2 [11]

Cg-Prp

H-ILENLLARSTNEDREGSIFDTGPIRRPKPRPRPRPEG-OH[11]

Cg-Defensin

H-GFGC(&^{1})PGNQLKC(&^{2})NNHC(&^{3})KSISC(&^{4})RAGYC(&^{1})DAATLWLRC(&^{2})TC(&^{3})TDC(&^{4})NGKK-OH [12]

PR-39

H-RRRPRPPYLPRPRPPPFFPPRLPPRIPPGFPPRFPPRFP-OH [13]

PR-26

H-Arg-Arg-Arg-Pro-Arg-Pro-Pro-Tyr-Leu-Pro-Arg-Pro-Arg-Pro-Pro-Pro-Phe-Phe-Pro- Pro-Arg-Leu-Pro-Pro-Arg-Ile-OH [13]

Pig Protegrin-3

H-Arg-Gly-Gly-Gly-Leu-Cys(&^{1})-Tyr-Cys(&^{2})-Arg-Arg-Arg-Phe-Cys(&^{2})-Val-Cys(&^{1})-Val-Gly-Arg-NH_2 [14]

L-Ser-Cecropin 6

H-GWLKKFGKKIERVGQHTRDATIQAIGVAQQAANVAATLKG-OH [15]

LSer-Def4

H-LTC(&^{1})NIDRSFC(&^{2})LAHC(&^{3})LLRGYKRGFC(&^{2})TVKKIC(&^{3})VC(&^{1})RH-OH[15]

PBD-1_{42}

H-KNIGNSVSC(&^{1})LRNKGVC(&^{2})MPGKC(&^{3})APKMKQIGTC(&^{2})GMPQVKC(&^{3})C(&^{1})KRK-OH [16]

PBD-1_{38}

H-NSVSC(&^{1})LRNKGVC(&^{2})MPGKC(&^{3})APKMKQIGTC(&^{2})GMPQVKC(&^{3})C(&^{1})KRK-OH [16]

Rabbit granulocytes peptides

NP1

H-VVCACRRALCLPRERRAGFCRIRGRIHPLCCRR-OH [17]

NP-2

H-VVCACRRALCLPLGRRAGFCRIRGRIHPLCCRR-OH [17]

NP-3a

H-GICACRRRFCPNSERFSGYCRVNGARYVRCCSRR-OH [17]

NP-5

H-VFCTCRGFLCGSGERASGSCTINGVRHTLCCR-OH [17]

Hst5

H-Asp-Ser-His-Ala-Lys-Arg-His-His-Gly-Tyr-Lys-Arg-Lys-Phe-His-Glu-Lys-His-His-Ser-His-Arg-Gly-Tyr-OH [7]

LL-37

H-LLGDFFRKSKEKIGKEFKRIVQRIKDFLRNLVPRTES-OH[18]

Cathelicidin-related AMP (CRAMP)

H-Gly-Leu-Leu-Arg-Lys-Gly-Gly-Glu-Lys-Ile-Gly-Glu-Lys-Leu-Lys-Lys-Ile-Gly-Gln-Lys-Ile-Lys-Asn-Phe-Phe-Gln-Lys-Leu-Val-Pro-Gln-Pro-Glu-Gln-OH [19]

Onc72

H-VDKPPYLPRPRPPROIYNO-NH_2 [19]

Onc110

H-VKPPYLPRPRPXRXJYNO-NH_2 [19]

Onc112

H-VKPPYLPRPRPPRrIYNr-NH_2 [19]

Api88

gu-ONRPVYIPRPRPPHPRL-NH_2 [19]

Api134

gu-ONNRPVYIPRPRPPHPOL-NH_2 [19]

Api137

gu-O-NNRPVYIPRPRPPHPRL-OH [19]

J, O, X and gu denote L-tert-leucine, L-ornithine, trans-4-hydroxy-L-proline and N,N,N´,N´-tetramethylguanidino, respectively[19]

Pig Protegrin-1

H-Arg-Gly-Gly-Arg-Leu-Cys(&^{1})-Tyr-Cys(&^{2})-Arg-Arg-Arg-Phe-Cys(&^{2})-Val-Cys(&^{1})-Val-Gly-Arg-NH_2 [14]

Bovine bactenecin

H-Arg-Leu-Cys(&^{1})-Arg-Ile-Val-Val-Ile-Arg-Val-Cys(&^{1})-Arg-OH [20]

Bovine indolicidin

H-Ile-Leu-Pro-Trp-Lys-Trp-Pro-Trp-Trp-Pro-Trp-Arg-Arg-NH_2 [21]

CAP11 is a homodimer of: H-GLRKKFRKTRKRIQKLGRKIGKTGRKVWKAWREYGQIPYPCRI-OH, joined with one disulfide bond [22]

Gallidermin

H-Ile-Ala-Ala(&^{1})-Lys-Phe-Leu-Ala(&^{1})-Abu(&^{2})-Pro-Gly-Ala(&^{2})-Ala-Lys-Dhb-Gly-Ala(&^{3})-Phe-Asn-Ala(&^{4})-Tyr-(&^{4})Ala(&^{3})-NH_2 [23]

Guinea pig defensin

H-Arg-Arg-Cys-Ile-Cys-Thr-Thr-Arg-Thr-Cys-Arg-Phe-Pro-Tyr-Arg-Arg-Leu-Gly-Thr-Cys-Ile-Phe-Gln-Asn-Arg-Val-Tyr-Thr-Phe-Cys-Cys-OH[24]

Nisin

H-Ile-Dhb-Ala(&^{1})-Ile-Dha-Leu-Ala(&^{1})-Abu(&^{2})-Pro-Gly-Ala(&^{2})-Lys-Abu(&^{3})-Gly-Ala-Leu-Met-Gly-Ala(&^{3})-Asn-Met-Lys-Abu(&^{4})-Ala-Abu(&^{5})-Ala(&^{4})-Asn-Ala(&^{5})-Ser-Ile-His-Val-Dha-Lys-OH

The different residues are linked by thioether bonds. Dha, dehydroalanine; Dhb, dehydrobutyrine; Ala-S-Ala, lanthionine; Abu-S-Ala; β-methyllanthionine.

<u>Bicereucin</u>; Bsjα

H-Gln-Arg-Ala-Dhb-Pro-Ala-Dhb-Pro-Ala-Dhb-Pro-Trp-Leu-Ile-Lys-Ala- $_{D}$Ala-Tyr-Val-Val- $_{D}$Ala-Gly-Ala-Gly-Val -$_{D}$Ala-Phe-Val-Ala -$_{D}$Ala-Tyr-Ile-Dhb-Val-Asn-OH [25]

Bsjβ

H-Gln-Arg-Ala-Dhb-Pro- Dhb-Leu-Ala-Dhb-Pro- Leu-Dhb-Pro-His-Dhb -Pro-Tyr-Ala-$_{D}$Abu-Tyr- Val-Val-$_{D}$Ala-Gly- Gly-Val-Val-$_{D}$Ala-Ala -Ile-$_{D}$Ala-Gly-Ile-Phe -R-Ala/S-Ala(&^{1})-Asn-Asn-Lys -Dhb-R-Ala(&^{1})-Leu-Gly-OH [25]

<u>Lichenicidin</u>; Lchα

OBu-Ile-Abu(&^{1})-Leu-Dha-Dhb-Ala(&^{1})-Ala-Ile-Leu-Ala(&^{2})-Lys-Pro-Leu-Gly-Asn-Asn-Gly-Tyr-Leu-Ala(&^{2})-Abu(&^{3})-Val-Abu(&^{4})-Lys-Glu-Ala(&^{3})-Met-Pro-Ser-Ala(&^{4})-Asn-OH [26]

Lchβ

OBu-Dhb-Pro-Ala-Dhb-Dhb-Ala(&^{1})-Dha-Trp-Thr-Ala(&^{1})-Ile-Dhb-Ala-Gly-Val-Dhb-Val-Ala(&^{2})-Ala-Ser-Leu-Ala(&^{2})-Pro-Abu(&^{3})-Dhb-Lys-Ala(&^{3})-Abu(&^{4})-Ser-Arg-Ala(&^{4})-OH [26]

2-oxobutyryl (OBu), lanthionine (Ala-S-Ala), and methyllanthionine (Abu-S-Ala).

<u>Lacticins</u>

LtnA1: H-Ala(&^{1})-Ala(&^{1})-Dbh-Asn-Dhb-Phe-$_{D}$Ala-Leu-Ala(&^{2})-Asp-Tyr-Trp-Gly-Asn-Asn-Gly-Ala-Trp-Ala(&^{2})-Abu(&^{3})-Leu-Abu(&^{4})-His-Glu-Ala(&^{3})-Met-Ala-Trp-Ala(&^{4})-Lys-OH [25, 27]

LtnA2

OBu-Dhb-Pro-Ala-Dhb-Pro-Ala-Ile-$_{D}$Ala-Ile-Leu-$_{D}$Ala-Ala-Tyr-Ile-Ala(&^{1})-Thr-Asn-Thr-Ala(&^{1})-Pro-Abu(&^{2})-Thr-Lys-Ala(&^{2})-Abu(&^{3})-Arg-Ala-Ala(&^{3})-OH [25, 27]

Dha, dehydroalanine; Dhb, dehydrobutyrine; Ala-S-Ala, lanthionine; Abu-S-Ala, β-methyllanthionine.

Plantaricin EF is a class-IIb two-peptide bacteriocin that consists of the 33-residue PlnE and the 34-residue PlnF peptides [28]

PlnE

H-Phe-Asn-Arg-Gly-Gly-Tyr-Asn-Phe-Gly-Lys-Ser-Val-Arg-His-Val-Val-Asp-Ala-Ile- Gly-Ser-Val-Ala-Gly-Ile-Arg-Gly-Ile-Leu-Lys-Ser-Ile-Arg-OH [28]

PlnF

H-Val-Phe-His-Ala-Tyr-Ser-Ala-Arg-Gly-Val-Arg-Asn-Asn-Tyr-Lys-Ser-Ala-Val-Gly-Pro-Ala-Asp-Trp-Val-Ile-Ser-Ala-Val-Arg-Gly-Phe-Ile-His-Gly-OH [28]

PlnJ

H-Gly-Ala-Trp-Lys-Asn-Phe-Trp-Ser-Ser-Leu-Arg-Lys-Gly-Phe-Tyr-Asp-Gly-Glu-Ala-Gly-Arg-Ala-Ile-Arg-Arg[29]

PlnK

H-Arg-Arg-Ser-Arg-Lys-Asn-Gly-Ile-Gly-Tyr-Ala-Ile-Gly-Tyr-Ala-Phe-Gly-Ala-Val-Glu- Arg-Ala-Val-Leu-Gly-Gly-Ser-Arg-Asp-Tyr-Asn-Lys[29]

L50A

H-MGAIAKLVAKFGWPIVKKYYKQIMQFIGEGWAINKIIEWIKKHI-OH [30]

L50B

H-MGAIAKLVTKFGWPLIKKFYKQIMQFIGQGWTIDQIEKWLKRH-OH [30]

Enterocin 7A

H-MGAIAKLVAKFGWPIVKKYYKQIMQFIGEGWAINKIIDWIKKHI-OH [31]

Enterocin 7B

H-MGAIAKLVAKFGWPFIKKFYKQIMQFIGQGWTIDQIEKWLKRH-OH [31]

Thuricin D-alpha

H-Gly-Met-Ala-Ala-Cys(&^{1})-Val-Ile-Gly-Cys(&^{2})-Ile-Gly-Ser-Cys(&^{3})-Val-Ile-Ser-Glu-Gly-Ile-Gly-Ser(&^{1})-Leu-Val-Gly-Thr(&^{2})-Ala-Phe-Thr(&^{3})-Leu-GlyOH [32]

Thuricin D-beta

H-Gly-Trp-Val-Ala-Cys(&^{1})-Val-Gly-Ala-Cys(&^{2})-Gly-Ser-Val-Cys Cys(&^{3})-Leu-Ala-Ser-Gly-Gly-Thr(&^{1})-Glu-Phe-Ala-Ala(&^{2})-Ala-Ser-Tyr(&^{3})-Phe-Leu-OH [32]

Thiols are linked to the α-C of the corresponding amino acids

Cbnx: H-Trp-Gly-Trp-Lys-Glu-Val-Val-Gln-Asn-Gly-Gln-Thr-Ile-Phe-Ser-Ala-Gly-Gln-Lys-Leu-Gly-Asn-Met-Val-Gly-Lys-Ile-Val-Pro-Leu-Pro-Phe-Gly-OH [33]

Cbny: H-Ser-Ala-Ile-Leu-Ala-Ile-Thr-Leu-Gly-Ile-Phe-Ala-Thr-Gly-Tyr-Gly-Met-Gly-Val-Gln-Lys-Ala-Ile-Asn-Asp-Arg-Arg-Lys-Lys-OH [33]

Plantaricin alpha: H-Lys-Cys-Lys-Trp-Trp-Asn-Ile-Ala-Cys-Asp-Leu-Gly-Asn-Asn-Gly-His-Val-Ala-Abu-Leu-Ala-His-Glu-Ala-Gln-Val-Ser-Ala-Asn-OH [34]

Plantaricin beta: Pyr-Gly-Ile-Pro-Cys-Dhb-Ile-Gly-Ala-Ala-Val-Ala-Ala-Ala-Ile-Ala-Val-Ala-Pro-Abu-Abu-Lys-Ala-Dha-Lys-Arg-Ala-Gly-Lys-Arg-Lys-Lys-OH [34]

Dermcidin (DCD)-derived peptides

DCD-1L

H- SSLLEKGLDGAKKAVGGLGKLGKDAVEDLESVGKGAVHDVKDVLDSVL[35]

DCD-1

H-SSLLEKGLDGAKKAVGGLGKLGKDAVEDLESVGKGAVHDVKDVLDSV-OH [35]

Bombinin

H-Ala-Lys-Gly-Leu-Gly-Ile-Gly-Gly-Ala-Leu-Leu-Ser-Ala-Ala-Lys-Val-Gly-Leu-Lys-Gly-Leu-Ala-Glu-His-Phe-Asn-NH_2 [36, 37]

Novel bombinin

H-Gly-Ile-Gly-Gly-Ala-Leu-Leu-Asn-Val-Gly-Lys-Val-Ala-Leu-Lys-Gly-Leu-Ala-Lys-Gly-Leu-Ala-Glu-His-Phe-Ala-Asn-NH_2[36, 37]

Bombinin HL

H-Leu-Leu-Gly-Pro-Val-Leu-Gly-Leu-Val-Ser-Asn-Val-Leu-Gly-Gly-Leu-Leu-NH_2 [36]

Protonectin

H-Ile-Leu-Gly-Thr-Ile-Leu-Gly-Leu-Leu-Lys-Gly-Leu-NH_2 [38, 39]

Protonectin (1–6)

H-Ile-Leu-Gly-Thr-Ile-Leu-NH_2 [39]

Abaecin (Bombus pascuorum)

H-FVPYNPPRPGQSKPFPSFPGHGPFNPKIQWPYPLPNPGH-OH[40]

Feleucin-BV1

H-Phe-Leu-Gly-Leu-Leu-Gly-Gly-Leu-Leu-NH_2[37]

feleucin (BV1-FLGLLGGLL or BV2-FLGLIGSLL) occupying positions 124–132. The name feleucins was coined to phonetically represent their structural features of possessing N-terminal Phe (F) residues and C-terminal Leu (L) amide residues. Note that the two feleucins differ in primary structure by conservative substitutions at positions

Gal7

H-FSRSPRYHMQCGYRGTFCTPGKCPYGNAYLGLCRPKYSCCRWL-OH [41]

Gal9

H-RGLPQDCERRGGFCSHKSCPPGIGRIGLCSKEDFCCRSRWYS-OH [41]

Moronecidin: H-Phe-Phe-His-His-Ile-Phe-Arg-Gly-Ile-Val-His-Val-Gly-Lys-Thr-Ile-His-(Lys/Arg)Leu-Val-Thr-Gly-Thr-NH_2 [42]

Bass hepcidin: (Gly-Cys(&[1])-Arg-Phe-Cys(&[2])-Cys(&[3])-Asn-Cys(&[4])-Cys(&[4])-Pro-Asn-Met-Ser-Gly-Cys(&[3])-Gly-Val-Cys(&[2])-Cys(&[1])-Arg-Phe) with eight putative [43]

Hymenoptaecin:

H-
QERGSIVIQGTKEGRNRPSLDIDYKQRVYDKNGMTGNAYGGVNIRPGQPTRQHAGFEFGKEYKNGFIRGQSEVQRGPGGRLSPYVGINGGFRF-OH [44]

SMAP29

H-Arg-Gly-Leu-Arg-Arg-Leu-Gly-Arg-Lys-Ile-Ala-His-Gly-Val-Lys-Lys-Tyr-Gly-Pro-Thr-Val-Leu-Arg-Ila-Ile-Arg-Ile-Ala-NH_2 [45]

OaBac5mini

H-Arg-Phe-Arg-Pro-Pro-Ile-Arg-Arg-Pro-Pro-Ile-Arg-Pro-Pro-Phe-Arg-Pro-Pro-Phe-Arg-Pro-Pro-Val-Arg-NH_2 [45]

OaBac7.5mini

H-Arg-Arg-Ile-Pro-Arg-Pro-Ile-Leu-Leu-Pro-Trp-Arg-Pro-Pro-Arg-Pro-Ile-Pro-Arg-Pro-Gln-Pro-Gln-Pro-Ile-Pro-Arg-Trp-Leu-OH [45]

<u>FLUCONAZOLE</u>

Cc-GRP

H-Gly-Asn-Glu-Gly-Gly-Gly-His-Gly-Gly-His-Gly-Gly-Tyr-Gly-Gly-Tyr-His-His-His- Gly-Gly-Gly-Gly-Gly-Gly-Tyr-Gly-Gly-Tyr-His-Gly-Gly-Gly-Gly-Ser-OH[46]

DS6

H-Cys-Lys-Tyr-Lys-Ala-Arg-Trp-Lys-Leu-Leu-OH [47]

KU4

H-Gly-Ile-Trp-Lys-Lys-Trp-Ile-Lys-Lys-Trp-Leu-Lys-Lys-Leu-Lys-Asn-Leu-Phe-NH_2[48]

Upnlys6

H-Gly-Val-Ile-Lys-Ala-Ala-Lys-Lys-Val-Val-Lys-Val-Leu-Lys-Lys-Leu-Phe-NH_2 [48]

KABT-AMP

H-Gly-Ile-Trp-Lys-Lys-Trp-Ile-Lys-Lys-Trp-Leu-Lys-Lys-Leu-Leu-Lys-Lys-Leu-Trp-Lys-Lys-Gly-OH [48]

Uperin 3.6

H-Gly-Val-Ile-Asp-Ala-Ala-Lys-Lys-Val-Val-Asn-Val-Leu-Lys-Asn-Leu-Phe-NH_2[48]

Upn-Lys5

H-Gly-Val-Ile-Lys-Ala-Ala-Lys-Lys-Val-Val-Lys-Val-Leu-Lys-Asn-Leu-Phe-NH_2 [48]

IB-367: H-Arg-Gly-Gly-Leu-Cys(&^{1})-Tyr-Cys(&^{2})-Arg-Gly-Arg-Phe-Cys(&^{2})-Val-Cys(&^{1})-Val-Gly-Arg-NH_2 [49]

Hepcidin 20

H-Ile-Cys(&)^{1}-Ile-Phe-Cys(&)^{2}-Cys(&)^{3}-Gly-Cys(&)^{4}-Cys(&)^{4}-His-Arg-Ser-Lys-Cys(&)^{3}-Gly-Met-Cys(&)^{2}-Cys(&)^{1}-Lys-Thr-OH [50].

Short lipopeptide palmitoyl

PAL-Lys-Lys-NH_2 [51]

Dodecapeptide

H-Arg-Trp-Trp-Arg-$_{D}$Trp-$_{D}$Phe-Ile-$_{D}$Phe-His-Trp-Arg-Trp-NH_2 [52]

RWWRWFIFH

H-Arg-Trp-Trp-Arg-$_{D}$Trp-$_{D}$Phe-Ile-$_{D}$Phe-His-NH_2 [53]

h-Lf1-11

H-Gly-Arg-Arg-Arg-Arg-Ser-Val-Gln-Trp-Cys-Ala-OH [55]

DsS3

H-Ala-Leu-Trp-Lys-Asn-Met-Leu-Lys-Gly-Ile-Gly-Lys-Leu-Ala-Gly-Lys-Ala-Ala-Leu-Gly-Ala-Val-Lys-Lys-Leu-Val-Gly-Ala-Glu-Ser-OH [56]

MUC7 12-mer-$_{L}$

H-Arg-Lys-Ser-Tyr-Lys-Cys-Leu-His-Lys-Arg-Cys-Arg-OH [57]

Tyrocidine A

&Val-Orn-Leu-$_{D}$Phe-Pro-Phe-$_{D}$Phe-Asn-Gln-Tyr& [58]

Tyrocidine B

&Val-Orn-Leu-$_{D}$Phe-Pro-Trp-$_{D}$Phe-Asn-Gln-Tyr& [58]

Tyrocidine C

&Val-Orn-Leu-$_{D}$Phe-Pro-Trp-$_{D}$Trp-Asn-Gln-Tyr& [58]

Plant defensin rHsAFP1

H-DGVKLCDVPSGTWSGHCGSSSKCSQQCKDREHFAYGGACHYQFPSVKCFCKRQC-OH [59]

HsLin06

H-Glu-His-Phe-Ala-Tyr-Gly-Gly-Ala-Xaa-His-Tyr-Gln-Phe-Pro-Ser-Val-Lys-Xaa-Phe-Xaa-Lys-Arg-Gln-Xaa-OH [59]

MSI-78

H-Gly-Ile-Gly-Lys-Phe-Leu-Lys-Lys-Ala-Lys-Lys-Phe-Gly-Lys-Ala-Phe-Val-Lys-Ile-Leu-Lys-Lys-NH_2 [60]

Cecropin B

H-KWKVFKKIEKMGRNIRNGIVKAGPAIAVLGEAKAL-NH_2 [60]

Dq-1319

H-Phe-Trp-Gly-Thr-Leu-Ala-Lys-Trp-Ala-Leu-Lys-OH [61]

Dq-1503

H-Phe-Trp-Gly-Thr-Leu-Ala-Lys-Trp-Ala-Leu-Lys-Ala-Ile-OH [61]

Dq-2562

H-Phe-Trp-Gly-Thr-Leu-Ala-Lys-Trp-Ala-Leu-Lys-Ala-Ile-Pro-Ala-Ala-Met-Gly-Met-Lys-Gln-Asn-Lys-OH [61]

Dq-3162

H-Gly-Leu-Lys-Asp-Trp-Trp-Asn-Lys-His-Lys-Asp-Lys-Ile-Val-Lys-Val-Val-Lys-Glu-Met-Gly-Lys-Ala-Gly-Ile-Asn-Ala-Ala-NH_2 [61]

Bacillomycin D

H-Ser-Thr-Asn-Tyr-Asn-Pro-Glu-OH [62]

KU1

H-Gly-Ile-Trp-Lys-Lys-Trp-Ile-Lys-Lys-Val-Val-Asn-Val-Leu-Lys-Asn-Leu-Phe-NH_2 [48]

KU2

H-Gly-Ile-Trp-Lys-Lys-Trp-Ile-Lys-Lys-Trp-Leu-Asn-Val-Leu-Lys-Asn-Leu-Phe-NH_2 [48]

KU3

H-Gly-Ile-Trp-Lys-Lys-Trp-Ile-Lys-Lys-Trp-Leu-Lys-Val-Leu-Lys-Asn-Leu-Phe-NH_2 [48]

KU4

H-Gly-Ile-Trp-Lys-Lys-Trp-Ile-Lys-Lys-Trp-Leu-Lys-Lys-Leu-Lys-Asn-Leu-Phe-NH_2 [48]

Upn-lys4

H-Gly-Val-Ile-Lys-Ala-Ala-Lys-Lys-Val-Val-Asn-Val-Leu-Lys-Asn-Leu-Phe-NH_2 [48]

Upn-lys5

H-Gly-Val-Ile-Lys-Ala-Ala-Lys-Lys-Val-Val-Lys-Val-Leu-Lys-Asn-Leu-Phe-NH_2 [48]

Upn-lys6

H-Gly-Val-Ile-Lys-Ala-Ala-Lys-Lys-Val-Val-Lys-Val-Leu-Lys-Lys-Leu-Phe-NH_2 [48]

Histatin 5

H-Asp-Ser-His-Ala-Lys-Arg-His-His-Gly-Tyr-Lys-Arg-Lys-Phe-His-Glu-Lys-His-His-Ser-His-Arg-Gly-Tyr-OH [63]

dhvar4

H-Lys-Arg-Leu-Phe-Lys-Lys-Leu-Leu-Phe-Ser-Leu-Arg-Lys-Tyr-OH [63]

dhvar5

H-Leu-Leu-Leu-Phe-Leu-Leu-Lys-Lys-Arg-Lys-Lys-Arg-Lys-Tyr-OH [63]

Short lactoferrin peptide Pep2

H-Phe-Lys-Cys-Arg-Arg-Trp-Gln-Trp-Arg-Met-OH [64]

Tachyplesin III

H-Lys-Trp-Cys($\&^1$)-Phe-Arg-Val-Cys($\&^2$)-Tyr-Arg-Gly-Ile-Cys($\&^2$)-Tyr-Arg-Lys-Cys($\&^1$)-Arg-NH_2 [65]

WLBU2

H-Arg-Arg-Trp-Val-Arg-Arg-Val-Arg-Arg-Trp-Val-Arg-Arg-Val-Val-Arg-Val-Val-Arg-Arg-Trp-Val-Arg-Arg-OH [66]

L12

H-Val-Arg-Ile-Ile-Trp-Ala-Val-Arg-Ile-Trp-Arg-Arg-OH [67]

D-LL-31

H-Leu-Leu-Gly-Asp-Phe-Phe-Arg-Lys-Ser-Lys-Glu-Lys-Ile-Gly-Lys-Glu-Phe-Lys-Arg-Ile-Val-Gln-Arg-Ile-Lys-Asp-Phe-Leu-Arg-Asn-Leu-OH [68]

LfcinB (20–25)

H-Arg-Arg-Trp-Gln-Trp-Arg-NH_2 [69]

LfcinB (20–30)

H-Arg-Arg-Trp-Gln-Trp-Arg-Met-Lys-Lys-Leu-Gly-NH_2 [69]

LfcinB (17–31)

H-Phe-Lys-Ala-Arg-Arg-Trp-Gln-Trp-Arg-Met-Lys-Lys-Leu-Gly-Ala-NH2 [69]

LfcinB

H-Phe-Lys-Cys-Arg-Arg-Trp-Gln-Trp-Arg-Met-Lys-Lys-Leu-Gly-Ala-Pro-Ser-Ile-Thr-Cys-Val-Arg-Arg-Ala-Phe-NH_2 [69]

L11W

H-Ile-Lys-Lys-Ile-Leu-Ser-Lys-Ile-Lys-Lys-Trp-Leu-Lys-NH_2 [70]

L12W

H-Ile-Lys-Lys-Ile-Leu-Ser-Lys-Ile- Lys-Lys-Leu-Trp-Lys-NH_2 [70]

I1WL5W

H-Trp-Lys-Lys-Ile-Trp-Ser-Lys-Ile-Lys-Lys-Leu-Leu-Lys-NH_2 [70]

I4WL5W

H-Ile-Lys-Lys-Trp-Trp-Ser-Lys-Ile-Lys-Lys-Leu-Leu-Lys-NH_2 [70]

SLAY-P1

H-Arg-Leu-Val-Arg-Ile-Leu-Val-Ser-Lys-Arg-Pro-Val-Ala-Ile-Lys-Pro-Tyr-Phe-Arg-Leu-OH[71]

Melimine

H-Thr-Leu-Ile-Ser-Trp-Ile-Lys-Asn-Lys-Arg-Lys-Gln-Arg-Pro-Arg-Val-Ser-Arg-Arg-Arg-Arg-Arg-Arg-Gly-Gly-Arg-Arg-Arg-Arg-OH [72]

Mel4

H-Lys-Asn-Lys-Arg-Lys-Arg-Arg-Arg-Arg-Arg-Arg-Gly-Gly-Arg-Arg-Arg-Arg-OH [72]

AamAP1 lysine

H-Phe-Leu-Phe-Lys-Leu-Ile-Pro-Lys-Ala-Ile-Lys-Lys-Leu-Ile-Ser-Lys-Phe-Lys-OH [73]

UP-5

H-Arg-B-Arg-B-Arg-COOH, where B represents biphenylalanine [74]

HYL

H-Gly-Ile-Met-Ser-Ser-Leu-Met-Lys-Lys-Leu-Ala-Ala-His-Ile-Ala-Lys-NH_2 [75]

I(LLKK)2I

H-Ile-Leu-Leu-Lys-Lys-Leu-Leu-Lys-Lys-Ile-NH_2 [76]

M(LLKK)2M

H-Met-Leu-Leu-Lys-Lys-Leu-Leu-Lys-Lys-Met-NH_2 [76]

W(LLKK)2W

H-Trp-Leu-Leu-Lys-Lys-Leu-Leu-Lys-Lys-Trp-NH_2 [76]

Plectasin

H-GFGCNGPWDEDDMQCHNHCKSIKGYKGGYCAKGGFVCKCY-OH [77]

Plectasin NZ2114:

H-GFGCNGPWNEDDLRCHNHCKSIKGYKGGYCAKGGFVCKCY-OH [78]

PL-5

Ac-Lys-Trp-Lys-Ser-Phe-Leu-Lys-Thr-Phe-Lys-Ser-Ala-Ala-Lys-Thr-Val- Leu -His-Thr-Ala-Leu-Lys-Ala-Ile-Ser-Ser-NH_2 [79]

PL-31

Ac-Lys-Trp-Lys-Ser-Phe-Leu-Lys-Thr-Phe-Lys-Ser-Leu-Lys-Lys-Thr-Val- Leu -His-Thr-Leu-Leu-Lys-Ala-Ile-Ser-Ser -NH_2 [79]

PL-32

Ac-Lys-Trp-Lys-Ser-Phe-Leu-Lys-Thr-Phe-Lys-Ser-Leu-Lys-Lys-Thr-Val-Leu-His-Thr-Leu-Leu-Lys-Ala-Ile-Ser-Ser-NH_2 [79]

PL-18

Ac-Phe-Lys-Lys-Leu-Lys-Lys-Leu-Phe-Ser-Lys-Leu-Trp-Asn-Trp-Lys-NH_2 [79]

PL-29

Ac-Phe-Lys-Lys-Leu-Lys-Lys-Leu-Phe-Ser-Lys-Leu-Phe-Ser-Phe-Lys-NH_2 [79]

PL-26

Ac- Lys-Lys-Val-Val-Phe-Lys-Val-Lys-Phe-Lys-Lys-$_{NH2}$ [79]

PRW4

Arg-Phe-Arg-Arg-Leu-Arg-Trp-Lys-Thr-Arg-Trp-Arg-Leu-Lys-Lys-Ile[80]

PMAP-36

H-GRFRRLRKKTRKRLKKIGKVLKWIPPIVGSIPLGCG-OH [80]

Coprisin

H-VTCDVLSFEAKGIAVNHSACALHCIALRKKGGSCQNGVCVCRN-NH_2 [81]

Pleurocidin

H-Gly-Trp-Gly-Ser-Phe-Phe-Lys-Lys-Ala-Ala-His-Val-Gly-Lys-His-Val-Gly -Lys-Ala-Ala-Leu-Thr-His-Tyr-Leu-NH_2 [82]

Protegrin 1: H-Arg-Gly-Gly-Arg-Leu-Cys(&[1])-Tyr-Cys(&[1])-Arg-Arg-Arg-Phe-Cys(&[1])-Val-Cys(&[1])-Val-Gly-Arg-OH [83]

Cathelicidin-BF

H-Lys-Phe-Phe-Arg-Lys-Leu-Lys-Lys-Ser-Val-Lys-Lys-Arg-Ala-Lys-Glu- Phe-Phe-Lys-Lys-Pro-Arg-Val-Ile-Gly-Val-Ser-Ile-Pro-Phe-OH [83]

BA250-C10

Arg-Trp-Arg-Trp-Arg-Trp-Lys-(C10) synthetic lipoAMP, a lipidated peptide with a C10-lipid chain attached to the C-terminus [84]

Bactenecin

H-Arg-Leu-Cys(&)[1]-Arg-Ile-Val-Val-Ile-Arg-Val-Cys(&)[1]-Arg-OH [85]

BP100

H-Lys-Lys-Leu-Phe-Lys-Lys-Ile-Leu-Lys-Tyr-Leu-NH_2 [86]

GA-K4

H-Phe-Leu-Lys-Trp-Leu-Phe-Lys-Trp-Ala-Lys-Lys-NH_2 [87]

HRAP1/HRAP2

Ac-Phe-Lys-Lys-Leu-Lys-Lys-Leu-Phe-Ser-Lys-Leu-Trp-Asn-Trp-Lys-NH_2 [88]

Parasin I

H-Lys-Gly-Arg-Gly-Lys-Gln-Gly-Gly-Lys-Val-Arg-Ala-Lys-Ala-Lys-Thr-Arg-Ser-Ser-OH [89]

Synoeca-MP peptide

H-Ile-Asn-Trp-Leu-Lys-Leu-Gly-Lys-Lys-Ile-Ile-Ala-Ser-Leu-NH_2 [90]

Bacillomycin D

&Asn-Pro-Glu-Ser-Thr-βXaa-Asp-Tyr& [91]

It is a mixture of two homologous lipopeptides: the lipid moiety consists of 3-amino-12-methyltridecanoic acid or 3-amino-12-methyltetradecanoic acid (βXaa)

Gramicidin-S

$(\&\text{Val-Orn-Leu-D-Phe-Pro}\&)_2$ [92]

Gramicidin A

HCO-L-Val-Gly-L-Ala-D-Leu-L-ala-D-Val-L-Val-D-Val-L-Trp-D-Leu-Trp-D-Teu-L-trp-D-leu-L-trp-$NHCH_2CH_2OH$ [93]

Thionin

H-KSCCRNTWARNCYNVCRLPGTISREICAKKCDCKIISGTTCPSDYPK-OH [94]

STF(1-37)

H-KIRTRRSQARKCSRGNGGGIRCPGGGIRLGGGSLIGR-OH [95]

D2A21

H-Phe-Ala-Lys-Lys-Phe-Ala-Lys-Lys-Phe-Lys-Lys-Phe-Ala-Lys-Lys-Phe-Ala-Lys-Phe-Ala-Phe-Ala-Phe-OH [96]

Clavanin A

H-Val-Phe-Gln-Phe-Leu-Gly-Lys-Ile-Ile-His-His-Val-Gly-Asn-Phe-Val-His-Gly-Phe-Ser-His-Val-Phe-NH_2 [97]

MBP-1

H-RSGRGECRRQCLRRHEGQPWETQECMRRCRRRG-OH [98]

Melittin

H-Gly-Ile-Gly-Ala-Val-Leu-Lys-Val-Leu-Thr-Thr-Gly-Leu-Pro-Ala-Leu-Ile-Ser-Trp-Ile-Lys-Arg-Lys-Arg-Gln-Gln-NH_2 [99]

Cecropin A

H-KWKLFKKIEKVGQNIRDGIIKAGPAVAVVGQAT-OH [100]

References

1. Zerweck, J., et al., *Molecular mechanism of synergy between the antimicrobial peptides PGLa and magainin 2.* Sci. Rep., 2017. **7**(1): p. 1-21.
2. Kabelka, I., et al., *Magainin 2 and PGLa in bacterial membrane mimics II: membrane fusion and sponge phase formation.* Biophysical journal, 2020. **118**(3): p. 612-623.
3. Capparelli, R., et al., *Synergistic antibacterial and anti-inflammatory activity of temporin A and modified temporin B in vivo.* PLoS One, 2009. **4**(9): p. No pp. given.
4. Bhunia, A., et al., *NMR structures and interactions of temporin-1Tl and temporin-1Tb with lipopolysaccharide micelles: mechanistic insights into outer membrane permeabilization and synergistic activity.* J Biol Chem, 2011. **286**(27): p. 24394-406.
5. Conlon, J.M., *Reflections on a systematic nomenclature for antimicrobial peptides from the skins of frogs of the family Ranidae.* Peptides, 2008. **29**(10): p. 1815-1819.

6. Ciandrini, E., et al., *Synergistic combinations of antimicrobial peptides against biofilms of methicillin-resistant Staphylococcus aureus (MRSA) on polystyrene and medical devices.* Journal of Global Antimicrobial Resistance, 2020. **21**: p. 203-210.
7. De Smet, K. and R. Contreras, *Human antimicrobial peptides: defensins, cathelicidins and histatins.* Biotechnology letters, 2005. **27**(18): p. 1337-1347.
8. Chairatana, P. and E.M. Nolan, *Human α-defensin 6: a small peptide that self-assembles and protects the host by entangling microbes.* Accounts of chemical research, 2017. **50**(4): p. 960-967.
9. Schmitt, P., et al., *The Antimicrobial Defense of the Pacific Oyster, Crassostrea gigas. How Diversity may Compensate for Scarcity in the Regulation of Resident/Pathogenic Microflora.* Front Microbiol, 2012. **3**: p. 160.
10. Schmitt, P., et al., *Expression, tissue localization and synergy of antimicrobial peptides and proteins in the immune response of the oyster Crassostrea gigas.* Dev. Comp. Immunol., 2012. **37**(3-4): p. 363-370.
11. Yannick, G., et al., *Oyster hemocytes express a proline-rich peptide displaying synergistic antimicrobial activity with a defensin.* Mol. Immunol., 2009. **46**(4): p. 516-522.
12. Gueguen, Y., et al., *Characterization of a defensin from the oyster Crassostrea gigas: recombinant production, folding, solution structure, antimicrobial activities, and gene expression.* Journal of Biological Chemistry, 2006. **281**(1): p. 313-323.
13. Shi, J., et al., *Antibacterial activity of a synthetic peptide (PR-26) derived from PR-39, a proline-arginine-rich neutrophil antimicrobial peptide.* Antimicrobial Agents and Chemotherapy, 1996. **40**(1): p. 115-121.
14. Lai, J.R., et al., *Design of non-cysteine-containing antimicrobial β-hairpins: Structure− activity relationship studies with linear protegrin-1 analogues.* Biochemistry, 2002. **41**(42): p. 12835-12842.
15. Poeppel, A.-K., et al., *Antimicrobial peptides expressed in medicinal maggots of the blow fly Lucilia sericata show combinatorial activity against bacteria.* Antimicrob. Agents Chemother., 2015. **59**(5): p. 2508-2514.
16. Shi, J., et al., *Porcine epithelial β-defensin 1 is expressed in the dorsal tongue at antimicrobial concentrations.* Infect. Immun., 1999. **67**(6): p. 3121-3127.
17. Selsted, M.E., et al., *Primary structures of six antimicrobial peptides of rabbit peritoneal neutrophils.* Journal of Biological Chemistry, 1985. **260**(8): p. 4579-4584.
18. Bedran, T.B.L., et al., *Synergistic anti-inflammatory activity of the antimicrobial peptides human beta-defensin-3 (hbD-3) and cathelicidin (LL-37) in a three-dimensional co-culture model of gingival epithelial cells and fibroblasts.* PLoS One, 2014. **9**(9): p. e106766/1-e106766/10, 10 pp.
19. Knappe, D., et al., *Insect-derived short proline-rich and murine cathelicidin-related antimicrobial peptides act synergistically on Gram-negative bacteria in vitro.* Future Med. Chem., 2016. **8**(10): p. 1035-1045.
20. Romeo, D., et al., *Structure and bactericidal activity of an antibiotic dodecapeptide purified from bovine neutrophils.* Journal of Biological Chemistry, 1988. **263**(20): p. 9573-9575.
21. Rozek, A., C.L. Friedrich, and R.E. Hancock, *Structure of the bovine antimicrobial peptide indolicidin bound to dodecylphosphocholine and sodium dodecyl sulfate micelles.* Biochemistry, 2000. **39**(51): p. 15765-15774.
22. Nagaoka, I., et al., *Synergistic actions of antibacterial neutrophil defensins and cathelicidins.* Inflamm Res, 2000. **49**(2): p. 73-9.
23. Bierbaum, G., et al., *The biosynthesis of the lantibiotics epidermin, gallidermin, Pep5 and epilancin K7.* Antonie Van Leeuwenhoek, 1996. **69**(2): p. 119-127.
24. Selsted, M.E. and S. Harwig, *Purification, primary structure, and antimicrobial activities of a guinea pig neutrophil defensin.* Infection and Immunity, 1987. **55**(9): p. 2281-2286.

25. Huo, L. and W.A. van der Donk, *Discovery and characterization of bicereucin, an unusual D-amino acid-containing mixed two-component lantibiotic.* J. Am. Chem. Soc., 2016. **138**(16): p. 5254-5257.
26. Shenkarev, Z.O., et al., *Isolation, Structure Elucidation, and Synergistic Antibacterial Activity of a Novel Two-Component Lantibiotic Lichenicidin from Bacillus licheniformis VK21.* Biochemistry, 2010. **49**(30): p. 6462-6472.
27. Cotter, P.D., et al., *Complete alanine scanning of the two-component lantibiotic lacticin 3147: generating a blueprint for rational drug design.* Mol. Microbiol., 2006. **62**(3): p. 735-747.
28. Ekblad, B., et al., *Structure–function analysis of the two-peptide bacteriocin plantaricin EF.* Biochemistry, 2016. **55**(36): p. 5106-5116.
29. Rogne, P., et al., *Three-dimensional structure of the two-peptide bacteriocin plantaricin JK.* Peptides, 2009. **30**(9): p. 1613-1621.
30. Izquierdo, E., et al., *Production of enterocins L50A, L50B, and IT, a new enterocin, by Enterococcus faecium IT62, a strain isolated from Italian ryegrass in Japan.* Antimicrobial agents and chemotherapy, 2008. **52**(6): p. 1917-1923.
31. Lohans, C.T., et al., *Solution Structures of the Linear Leaderless Bacteriocins Enterocin 7A and 7B Resemble Carnocyclin A, a Circular Antimicrobial Peptide.* Biochemistry, 2013. **52**(23): p. 3987-3994.
32. Sit, C.S., et al., *The 3D Structure of Thuricin CD, a Two-Component Bacteriocin with Cysteine Sulfur to α-Carbon Cross-links.* J. Am. Chem. Soc., 2011. **133**(20): p. 7680-7683.
33. Acedo, J.Z., et al., *Identification and three-dimensional structure of carnobacteriocin XY, a class IIb bacteriocin produced by Carnobacteria.* FEBS Lett, 2017. **591**(10): p. 1349-1359.
34. Holo, H., et al., *Plantaricin W from Lactobacillus plantarum belongs to a new family of two-peptide lantibiotics.* Microbiology (Reading, U. K.), 2001. **147**(3): p. 643-651.
35. Steffen, H., et al., *Naturally processed dermcidin-derived peptides do not permeabilize bacterial membranes and kill microorganisms irrespective of their charge.* Antimicrobial agents and chemotherapy, 2006. **50**(8): p. 2608-2620.
36. Xiang, J., et al., *The synergistic antimicrobial effects of novel bombinin and bombinin H peptides from the skin secretion of Bombina orientalis.* Biosci. Rep., 2017. **37**(5): p. BSR20170967.
37. Bai, B., et al., *Feleucins: novel bombinin precursor-encoded nonapeptide amides from the skin secretion of Bombina variegata.* Biomed Res Int, 2014. **2014**: p. 671362.
38. Wang, K., et al., *Antimicrobial peptide protonectin disturbs the membrane integrity and induces ROS production in yeast cells.* Biochimica et Biophysica Acta (BBA)-Biomembranes, 2015. **1848**(10): p. 2365-2373.
39. Baptista-Saidemberg, N.B., et al., *Protonectin (1–6): A novel chemotactic peptide from the venom of the social wasp Agelaia pallipes pallipes.* Toxicon, 2010. **56**(6): p. 880-889.
40. Rahnamaeian, M., et al., *Insect antimicrobial peptides show potentiating functional interactions against Gram-negative bacteria.* Proceedings of the Royal Society B: Biological Sciences, 2015. **282**(1806): p. 20150293.
41. Milona, P., et al., *The chicken host peptides, gallinacins 4, 7, and 9 have antimicrobial activity against Salmonella serovars.* Biochem. Biophys. Res. Commun., 2007. **356**(1): p. 169-174.
42. Lauth, X., et al., *Discovery and characterization of two isoforms of moronecidin, a novel antimicrobial peptide from hybrid striped bass.* Journal of Biological Chemistry, 2002. **277**(7): p. 5030-5039.
43. Shike, H., et al., *Bass hepcidin is a novel antimicrobial peptide induced by bacterial challenge.* European Journal of Biochemistry, 2002. **269**(8): p. 2232-2237.
44. Xu, P., M. Shi, and X.-x. Chen, *Antimicrobial peptide evolution in the Asiatic honey bee Apis cerana.* Plos one, 2009. **4**(1): p. e4239.

45. Anderson, R.C. and P.-L. Yu, *Factors affecting the antimicrobial activity of ovine-derived cathelicidins against E. coli O157:H7.* Int. J. Antimicrob. Agents, 2005. **25**(3): p. 205-210.
46. Zottich, U., et al., *An antifungal peptide from Coffea canephora seeds with sequence homology to glycine-rich proteins exerts membrane permeabilization and nuclear localization in fungi.* Biochimica et Biophysica Acta (BBA)-General Subjects, 2013. **1830**(6): p. 3509-3516.
47. Singh, K., et al., *DS6: anticandidal, antibiofilm peptide against Candida tropicalis and exhibit synergy with commercial drug.* Journal of Peptide Science, 2017. **23**(3): p. 228-235.
48. Lum, K.Y., et al., *Activity of Novel Synthetic Peptides against Candida albicans.* Sci. Rep., 2015. **5**: p. 9657.
49. Simonetti, O., et al., *In vitro activity of the protegrin IB-367 alone and in combination compared with conventional antifungal agents against dermatophytes.* Mycoses, 2014. **57**(4): p. 233-239.
50. Hunter, H.N., et al., *The Solution Structure of Human Hepcidin, a Peptide Hormone with Antimicrobial Activity That Is Involved in Iron Uptake and Hereditary Hemochromatosis* 210.* Journal of Biological Chemistry, 2002. **277**(40): p. 37597-37603.
51. Kamysz, E., et al., *In vitro activity of the lipopeptide PAL-Lys-Lys-NH2, alone and in combination with antifungal agents, against clinical isolates of Candida spp.* Peptides, 2011. **32**(1): p. 99-103.
52. Duggineni, S., et al., *A novel dodecapeptide from a combinatorial synthetic library exhibits potent antifungal activity and synergy with standard antimycotic agents.* Int. J. Antimicrob. Agents, 2007. **29**(1): p. 73-78.
53. Kumar, M., et al., *Identification of a novel antifungal nonapeptide generated by combinatorial approach.* International journal of antimicrobial agents, 2005. **25**(4): p. 313-320.
54. Mora-Navarro, C., et al., *Synthetic antimicrobial β-peptide in dual-treatment with fluconazole or ketoconazole enhances the in vitro inhibition of planktonic and biofilm Candida albicans.* Journal of Peptide Science, 2015. **21**(12): p. 853-861.
55. Lupetti, A., et al., *Candidacidal activities of human lactoferrin peptides derived from the N terminus.* Antimicrobial Agents and Chemotherapy, 2000. **44**(12): p. 3257-3263.
56. Mor, A., K. Hani, and P. Nicolas, *The vertebrate peptide antibiotics dermaseptins have overlapping structural features but target specific microorganisms.* Journal of Biological Chemistry, 1994. **269**(50): p. 31635-31641.
57. Wei, G.-X. and L.A. Bobek, *Human salivary mucin MUC7 12-mer-L and 12-mer-D peptides: antifungal activity in saliva, enhancement of activity with protease inhibitor cocktail or EDTA, and cytotoxicity to human cells.* Antimicrobial agents and chemotherapy, 2005. **49**(6): p. 2336-2342.
58. Troskie, A.M., et al., *Synergistic activity of the tyrocidines, antimicrobial cyclodecapeptides from Bacillus aneurinolyticus, with amphotericin B and caspofungin against Candida albicans biofilms.* Antimicrobial agents and chemotherapy, 2014. **58**(7): p. 3697-3707.
59. Vriens, K., et al., *Synergistic activity of the plant defensin HsAFP1 and caspofungin against Candida albicans biofilms and planktonic cultures.* PloS one, 2015. **10**(8): p. e0132701.
60. Denardi, L.B., et al., *Activity of MSI-78, h-Lf1-11 and cecropin B antimicrobial peptides alone and in combination with voriconazole and amphotericin B against clinical isolates of Fusarium solani.* Journal of Medical Mycology, 2021. **31**(2): p. 101119.
61. Dodou Lima, H.V., C.S. de Paula Cavalcante, and G. Rádis-Baptista, *Antifungal in vitro activity of pilosulin-and ponericin-like peptides from the giant ant Dinoponera quadriceps and synergistic effects with antimycotic drugs.* Antibiotics, 2020. **9**(6): p. 354.
62. Olfa, T., et al., *Synergistic fungicidal activity of the lipopeptide bacillomycin D with amphotericin B against pathogenic Candida species.* FEMS Yeast Research, 2015. **15**(4): p. fov022.

63. van't, H.W., et al., *Synergistic effects of low doses of histatin 5 and its analogues on amphotericin B anti-mycotic activity.* Antonie Van Leeuwenhoek, 2000. **78**(2): p. 163-9.
64. Tanida, T., et al., *Antimicrobial peptides enhance the candidacidal activity of antifungal drugs by promoting the efflux of ATP from Candida cells.* Journal of Antimicrobial Chemotherapy, 2006. **57**(1): p. 94-103.
65. Simonetti, O., et al., *In vitro activity of Tachyplesin III alone and in combination with terbinafine against clinical isolates of dermatophytes.* Peptides, 2009. **30**(10): p. 1794-1797.
66. Ryder, M.P., et al., *Binding interactions of bacterial lipopolysaccharide and the cationic amphiphilic peptides polymyxin B and WLBU2.* Colloids and Surfaces B: Biointerfaces, 2014. **120**: p. 81-87.
67. Xiong, F., et al., *Effects of the antimicrobial peptide L12 against multidrug-resistant Staphylococcus aureus.* Mol. Med. Rep., 2019. **19**(4): p. 3337-3344.
68. Wongkaewkhiaw, S., et al., *D-LL-31 in combination with ceftazidime synergistically enhances bactericidal activity and biofilm destruction in Burkholderia pseudomallei.* Biofouling, 2019. **35**(5): p. 573-584.
69. Vargas-Casanova, Y., et al., *Synergistic bactericide and antibiotic effects of dimeric, tetrameric, or palindromic peptides containing the RWQWR motif against Gram-positive and Gram-negative strains.* RSC Adv., 2019. **9**(13): p. 7239-7245.
70. Shang, D., et al., *Synergistic Antibacterial Activity of Designed Trp-Containing Antibacterial Peptides in Combination With Antibiotics Against Multidrug-Resistant Staphylococcus epidermidis.* Front Microbiol, 2019. **10**: p. 2719.
71. Liu, Y., et al., *Antagonizing Vancomycin Resistance in Enterococcus by Surface Localized Antimicrobial Display-Derived Peptides.* ACS Infect. Dis., 2019: p. Ahead of Print.
72. Kampshoff, F., M.D.P. Willcox, and D. Dutta, *A pilot study of the synergy between two antimicrobial peptides and two common antibiotics.* Antibiotics 2019. **8**(2): p. 60.
73. Almaaytah, A., A. Abualhaijaa, and O. Alqudah, *The evaluation of the synergistic antimicrobial and antibiofilm activity of AamAP1-Lysine with conventional antibiotics against representative resistant strains of both Gram-positive and Gram-negative bacteria.* Infect. Drug Resist., 2019. **12**: p. 1371-1380.
74. Almaaytah, A., et al., *Antimicrobial and antibiofilm activity of UP-5, an ultrashort antimicrobial peptide designed using only arginine and biphenylalanine.* Pharmaceuticals, 2018. **11**(1): p. 3/1-3/18.
75. Nesuta, O., et al., *Antimicrobial Peptide from the Wild Bee Hylaeus signatus Venom and Its Analogues: Structure-Activity Study and Synergistic Effect with Antibiotics.* J. Nat. Prod., 2016. **79**(4): p. 1073-1083.
76. Khara, J.S., et al., *Designing α-helical peptides with enhanced synergism and selectivity against Mycobacterium smegmatis: Discerning the role of hydrophobicity and helicity.* Acta Biomater., 2015. **28**: p. 99-108.
77. Mygind, P.H., et al., *Plectasin is a peptide antibiotic with therapeutic potential from a saprophytic fungus.* Nature, 2005. **437**(7061): p. 975-980.
78. Li, Z., et al., *Research advances on plectasin and its derivatives as new potential antimicrobial candidates.* Process Biochemistry, 2017. **56**: p. 62-70.
79. Feng, Q., et al., *Functional synergy of α-helical antimicrobial peptides and traditional antibiotics against Gram-negative and Gram-positive bacteria in vitro and in vivo.* Eur. J. Clin. Microbiol. Infect. Dis., 2015. **34**(1): p. 197-204.
80. Wang, Z., et al., *Synergistic interaction of PMAP-36 and PRW4 with aminoglycoside antibiotics and their antibacterial mechanism.* World J. Microbiol. Biotechnol., 2014. **30**(12): p. 3121-3128.
81. Hwang, I.-s., et al., *Synergistic Effect and Antibiofilm Activity Between the Antimicrobial Peptide Coprisin and Conventional Antibiotics Against Opportunistic Bacteria.* Curr. Microbiol., 2013. **66**(1): p. 56-60.

82. Choi, H. and D.G. Lee, *Antimicrobial peptide pleurocidin synergizes with antibiotics through hydroxyl radical formation and membrane damage, and exerts antibiofilm activity.* Biochim. Biophys. Acta, Gen. Subj., 2012. **1820**(12): p. 1831-1838.
83. Liu, Y.-F., et al., *Antibacterial Activity, Cytotoxicity and Mechanisms of action of Cathelicidin Peptides against Enteric Pathogens in Weaning Piglets.* Int. J. Pept. Res. Ther., 2011. **17**(3): p. 175-184.
84. de Gier, M.G., et al., *Synergistic activity of a short lipidated antimicrobial peptide (lipoAMP) and colistin or tobramycin against Pseudomonas aeruginosa from cystic fibrosis patients.* MedChemComm, 2016. **7**(1): p. 148-156.
85. Wu, M. and R.E. Hancock, *Interaction of the cyclic antimicrobial cationic peptide bactenecin with the outer and cytoplasmic membrane.* Journal of Biological Chemistry, 1999. **274**(1): p. 29-35.
86. Cabrefiga, J. and E. Montesinos, *Lysozyme enhances the bactericidal effect of BP100 peptide against Erwinia amylovora, the causal agent of fire blight of rosaceous plants.* BMC Microbiol., 2017. **17**: p. 39/1-39/10.
87. Kang, S.-J., H.-Y. Ji, and B.-J. Lee, *Anticancer activity of undecapeptide analogues derived from antimicrobial peptide, Brevinin-1EMa.* Arch. Pharmacal Res., 2012. **35**(5): p. 791-799.
88. Zhao, J., et al., *In vitro characterization of the rapid cytotoxicity of anticancer peptide HPRP-A2 through membrane destruction and intracellular mechanism against gastric cancer cell lines.* PLoS One, 2015. **10**(9): p. e0139578.
89. Park, I.Y., et al., *Parasin I, an antimicrobial peptide derived from histone H2A in the catfish, Parasilurus asotus.* FEBS letters, 1998. **437**(3): p. 258-262.
90. Dantas, E.M.G.L., et al., *Synergistic activity of chlorhexidine and synoeca-MP peptide against Pseudomonas aeruginosa.* J. Cell. Physiol., 2019. **234**(9): p. 16068-16079.
91. PEYPOUX, F., et al., *Structure of bacillomycin D, a new antibiotic of the iturin group.* European journal of biochemistry, 1981. **118**(2): p. 323-327.
92. Nagamurthi, G. and S. Rambhav, *Gramicidin-S: Structure-activity relationship.* Journal of biosciences, 1985. **7**(3): p. 323-329.
93. Wallace, B., *Structure of gramicidin A.* Biophysical journal, 1986. **49**(1): p. 295-306.
94. Vila-Perelló, M., et al., *Synthetic and structural studies on Pyrularia pubera thionin: a single-residue mutation enhances activity against Gram-negative bacteria.* FEBS letters, 2003. **536**(1-3): p. 215-219.
95. D'Este, F., et al., *Antimicrobial and host cell-directed activities of Gly/Ser-rich peptides from salmonid cathelicidins.* Fish Shellfish Immunol., 2016. **59**: p. 456-468.
96. Ballweber, L., et al., *In vitro microbicidal activities of cecropin peptides D2A21 and D4E1 and gel formulations containing 0.1 to 2% D2A21 against Chlamydia trachomatis.* Antimicrobial agents and chemotherapy, 2002. **46**(1): p. 34-41.
97. Duay, S.S., et al., *Molecular dynamics investigation into the effect of zinc (II) on the structure and membrane interactions of the antimicrobial peptide Clavanin A.* The Journal of Physical Chemistry B, 2019. **123**(15): p. 3163-3176.
98. Slavokhotova, A.A. and E.A. Rogozhin, *Defense peptides from the α-hairpinin family are components of plant innate immunity.* Frontiers in Plant Science, 2020. **11**: p. 465.
99. Deng, Z., et al., *Lipid-specific interactions determine the organization and dynamics of membrane-active peptide melittin.* Soft Matter, 2020. **16**(14): p. 3498-3504.
100. Merrifield, R., L. Vizioli, and H. Boman, *Synthesis of the antibacterial peptide cecropin A (1-33).* Biochemistry, 1982. **21**(20): p. 5020-5031.
